# The elusive parasite: comparing macroscopic, immunological, and genomic approaches to identifying malaria in human skeletal remains from Sayala, Egypt (third to sixth centuries AD)

**DOI:** 10.1007/s12520-021-01350-z

**Published:** 2021-06-14

**Authors:** Alvie Loufouma Mbouaka, Michelle Gamble, Christina Wurst, Heidi Yoko Jäger, Frank Maixner, Albert Zink, Harald Noedl, Michaela Binder

**Affiliations:** 1 Institute of Specific Prophylaxis and Tropical Medicine, Center for Pathophysiology, Infectiology and Immunology, Medical University of Vienna, Kinderspitalgasse 15, 1090 Vienna, Austria; 2 Bioarchaeology Department, Austrian Archaeological Institute at the Austrian Academy of Sciences, Franz Klein-Gasse 1, 1190 Vienna, Austria; 3 Present Address: Heritage and Archaeological Research Practice, 101 Rose Street South Lane, EH2 3JG Edinburgh, Scotland; 4 Institute for Mummy Studies, EURAC Research, Viale Druso 1, 39100 Bolzano, Italy; 5 Present Address: Malaria Research Initiative Bandarban, Vienna, Austria; 6 Present Address: Planen und Bauen im Bestand, Novetus, Belvederegasse 41, 1040 Vienna, Austria

**Keywords:** Malaria, Immunoassays, Shotgun-capture sequencing, aDNA, Palaeopathology

## Abstract

Although malaria is one of the oldest and most widely distributed diseases affecting humans, identifying and characterizing its presence in ancient human remains continue to challenge researchers. We attempted to establish a reliable approach to detecting malaria in human skeletons using multiple avenues of analysis: macroscopic observations, rapid diagnostic tests, and shotgun-capture sequencing techniques, to identify pathological changes, *Plasmodium* antigens, and *Plasmodium* DNA, respectively. Bone and tooth samples from ten individuals who displayed skeletal lesions associated with anaemia, from a site in southern Egypt (third to sixth centuries AD), were selected. *Plasmodium* antigens were detected in five of the ten bone samples, and traces of *Plasmodium* aDNA were detected in six of the twenty bone and tooth samples. There was relatively good synchronicity between the biomolecular findings, despite not being able to authenticate the results. This study highlights the complexity and limitations in the conclusive identification of the *Plasmodium* parasite in ancient human skeletons. Limitations regarding antigen and aDNA preservation and the importance of sample selection are at the forefront of the search for malaria in the past. We confirm that, currently, palaeopathological changes such as cribra *orbitalia* are not enough to be certain of the presence of malaria. While biomolecular methods are likely the best chance for conclusive identification, we were unable to obtain results which correspond to the current authentication criteria of biomolecules. This study represents an important contribution in the refinement of biomolecular techniques used; also, it raises new insight regarding the consistency of combining several approaches in the identification of malaria in past populations.

**Supplementary Information:**

The online version contains supplementary material available at 10.1007/s12520-021-01350-z.

## Introduction

### Malaria in the past

Discussions of disease in the past must take a multi-disciplinary approach, relying on archaeological contextual information, material culture studies, and, most importantly, analyses of human remains (i.e. Turner and Klaus 2016; Zuckerman et al. 2012). Palaeopathological analyses have formed an integral part of understanding the presence and prevalence of diseases in the past, primarily through macroscopic identification of changes to the skeleton. However, there are limitations to the diagnoses available as not all diseases leave their marks on the skeleton and even when present may not be pathognomonic. Thus, the development and integration of the analysis of ancient biomolecules associated with targeted diseases has provided a new tool to confirm the presence of pathogens in ancient human skeletal material and, moreover, can provide insight regarding the evolutionary story of a disease. Ancient DNA is the primary method explored here, but proteomics and isotopic analyses are quickly gaining traction as methods improve (i.e. Brown and Brown 2011; Dutour 2016; see Spyrou et al. 2019 for a review).

Shortly after the first recovery and sequencing of ancient DNA (Pääbo 1985, 1989; Pääbo et al. 1989, 2004), scientists began exploring this tool for evaluating the evolutionary history, the origins, the distribution, and the identification of ancient pathogens and diseases effecting humans (i.e. Donoghue et al. 1998; Marciniak 2016; Spyrou et al. 2019). As the methods and techniques of aDNA analysis have been refined, and continue to improve, it is now possible to identify more elusive pathogens and to establish the phylogeographic patterns of ancient diseases such as malaria, caused by *Plasmodium* parasites (regarding malaria — see Nerlich et al. 2008 and Lalremruata et al. 2013 for mummified tissue; Sallares and Gomzi 2001 and Marciniak et al. 2016, 2018 for skeletal material; and Gelabert et al. 2016 from blood on glass slides).

According to the World Health Organization (WHO), malaria is a life-threatening disease in humans caused by the protozoan parasite of the genus *Plasmodium* (five species are known to infect humans: *falciparum*, *vivax*, *ovale*, *malariae*, and *knowlesi*; with many other species infecting a variety of other animals). The parasite is transmitted to humans through the bite of infected female Anopheles mosquitoes. In 2018, an estimated 228 million cases of malaria worldwide were reported (WHO 2019). The disease has a wide global distribution and is a significant health burden within many tropical regions of the world (Murray et al. 2012; Caminade et al. 2014). *Plasmodium falciparum* is the most virulent and may cause severe malarial conditions; *P*. *falciparum*, along with *Plasmodium vivax*, represents the majority of malaria infections worldwide. The *Plasmodium* parasite is injected into human skin by an infected Anopheles mosquito and invades the bloodstream, settles in the liver, replicates, and infects the erythrocyte. There, they differentiate mainly into trophozoites and schizonts and a smaller portion into sexual stages (gametocytes). In *P*. *falciparum* infections, the later developmental stages (trophozoites and schizonts) are sequestered in the lumen of small vessels via their adhesion to microvascular endothelial cells, potentially leading to severe disease (Scherf et al. 2001; Farfour et al. 2012; Obaldia 3rd et al. 2018). It has been shown that the density of immature gametocytes is tenfold more on bone marrow smears than on peripheral blood smears (Alano 2017; Smalley et al. 1981), but their exact location is still unknown (Farfour et al. 2012; Obaldia 3rd et al. 2018). Bones and teeth can possibly carry genetic information for up to a million years (Hofreiter et al. 2015; van der Valk et al. 2021), and they are also rich in biomolecules that can persist for up to 3.4 million years and thus are an excellent source of ancient proteins (Demarchi et al. 2016: 3). Therefore, skeletal tissue is seemingly ideal for investigating blood-borne diseases, such as malaria, in past populations.

Malaria has plagued humans for millennia; however, research seems to indicate that agricultural intensification and the development of more permanent settlements lead to the increase in malaria infections (Hedrick 2012; Lalremruata et al. 2013). Agriculture requires areas of standing water as reserves to ensure crop production, and people began to live more densely, creating a target-rich environment for the mosquitoes which were finding new places to breed in the standing water, thus creating an ideal situation for the spread of malaria through human communities. While the identification of the parasite causing malaria is relatively recent (in 1907 French doctor, Charles Louis Alphonse Laveran, was awarded the Nobel Prize for identifying the protozoan parasite (Arrow et al. 2004: 128)), the characteristic symptoms of the disease were described in texts dating as far back as 2700 BC, in the Nei Ching (Chinese Canon of Medicine), which recorded a relationship between deadly periodic fevers and the enlargement of the spleen (Bruce-Chwatt 1988; Garcia 2010; Oaks Jr. et al. 1991). It is believed to appear in several Classical Greek and Roman texts, with the ancient populations associating the intermittent fevers with a marshy environment (Hempelmann and Krafts 2013; Sallares 2002). Researchers believe that *P*. *vivax* was present and recorded in the Eastern Mediterranean in ancient texts by the fifth century BC (Sallares 2002: 13). The arrival and transmission of *Plasmodium falciparum* from Africa into the Mediterranean is less certain, with conclusions relying on proxy-indications such as skeletal changes associated with anaemia (i.e. Angel 1966). Therefore, while the exact route taken by the *Plasmodium* parasites cannot be determined, it is clear from text references to intermittent fevers that after its arrival in Mediterranean countries, it spread through much of Southern Europe by the twelfth century (Garcia 2010). Within the twentieth century, the environmental and social changes in Europe due to the rapid development of the economy lead to the decline and eradication of the disease in this part of the world: swamps were drained, the urban areas were expanded, and the healthcare systems were improved (Hay et al. 2004; Piperaki and Daikos 2016).

It has only been rather recently that the evolutionary origins of human *Plasmodium falciparum* and *Plasmodium vivax* have been determined, though it is still unclear how or why it was able to cross from apes to humans (Loy et al. 2016: 9). While research has primarily focused on the transmission, diagnosis, treatment, and prevention of malaria, it is still poorly understood in regard to its evolutionary history (though this is rapidly changing with studies such as Loy et al.*,* 2016, and Galaway et al.*,* 2019) and it is difficult to systematically identify victims of this disease in ancient human remains. This is despite the fact that malaria has had an impact on recent human evolution through the emergence of inherited anaemias, such as the sickle cell disease or thalassemia, which are directly associated as a genetic response to malaria in past populations (Carter and Mendis 2002: 570; Hartl 2004; Kwiatkowski 2005: 185; James et al. 2019: 540-541). Therefore, the longevity and virulence of this parasite and subsequent disease within human populations make it essential to understand its impact on past populations, and this can only be achieved with consistent and reliable methods of identification in skeletal material.

### Identifying malaria in ancient human remains

Currently, there are several approaches used to identify malaria in ancient human remains (for synopses, see Bianucci et al. 2015; Nerlich 2016; and Setzer 2014): (1) the analysis of the skeletal lesions associated with anaemia, such as porotic hyperostosis and *cribra orbitalia* as an indication of chronic anaemia and a proxy for the presence of malaria (i.e. Angel 1966; Gowland and Garnsey 2010; Gowland and Western 2012; Smith-Guzmán 2015a, 2015b; Soren et al. 1995; Tayles 1996); (2) conventional microscopy and analysis of old slides treated with traditional staining techniques (Gelabert et al. 2016); (3) scanning electron microscopy and mass spectrometry to identify hemozoin (also known as malarial pigment), an insoluble by-product of the digestion of haemoglobin by the *Plasmodium* parasite during infection of the red blood cell (RBCs) and which can be localised in the skeleton (Cox 2018; Lee et al. 2017); (4) rapid diagnostic tests (RDTs), which are immunochromatographic tests primed with antibodies to detect and identify *Plasmodium* antigens (i.e. Al-Khafif et al. 2018; Bianucci et al. 2008; Bianucci et al. 2015; Fornaciari et al. 2010; Miller et al. 1994; Rabino Massa et al. 2000); (5) the analysis of *Plasmodium* DNA extracted from ancient human remains or from old blood slides (for human tissue: Lalremruata et al. 2013; Marciniak et al. 2016; Nerlich et al. 2008; Sallares and Gomzi 2001; for blood slides: Gelabert et al. 2016). Ideally, a combination of methods should be applied, to unambiguously prove the presence of *Plasmodium* in ancient human remains. However, each approach has challenges which complicate the conclusive identification of this parasite and our ability to determine the true prevalence and impact of malaria on past populations.

We used three different approaches on the same set of samples to generate discussion on the best practice methods for identifying malaria in the past. A sample of 10 skeletons from Sayala, Egypt, dating to the late third to mid sixth centuries AD, were re-examined in a macroscopic palaeopathological analysis, and a bone and tooth sample were selected from each individual to test immunologically and genetically for evidence of the presence of malaria. The immunological test consisted of using rapid diagnostic tests which will be described in brief below but has been published more extensively in Loufouma Mbouaka et al. (2020). The ancient DNA approach combines both a shotgun-based and capture-sequencing-based screening of the samples for *Plasmodium* DNA, using a similar approach as described by Marciniak et al. (2016). The purpose of this paper is to discuss not only the current possibilities but also limitations of providing evidence for malaria within this population.

#### Skeletal morphological changes and malaria

Physiological stress during life, through disease, trauma, dietary deficiencies, and a multitude of cultural, environmental, and economic causes, can be reflected on the human skeleton in the form of observable morphological changes (Agarwal 2016; Larsen 2018; Ortner 2003). Malaria in past populations has typically been interpreted through correlating skeletal indicators of chronic anaemia as a proxy, along with ecological and historical data (i.e. Gowland and Western 2012; Setzer 2014: 97). This is based on the belief that porotic hyperostosis (PH) and *cribra orbitalia* (CO) are lesions associated with anaemia and therefore possibly malaria (Angel 1966; Stuart-Macadam 1987, 1992). Both are amongst the most commonly reported pathological changes reported in palaeopathological analyses (see Walker et al. 2009: 109). This paper is not the place for a discussion on the potential causes of PH and CO (see for example Brickley 2018; Oxenham and Cavill 2010; Rivera and Mirazón Lahr 2017; Walker et al. 2009) or for the debate on whether there is a link between these two lesions (see, for example Brickley 2018; Cole and Waldron 2019; Hens et al. 2019; Stuart-Macadam 1987, 1992). Previous research on malaria has used these two lesions for their association with anaemia (i.e. Angel 1966; Gowland and Garnsey 2010; Gowland and Western 2012; Smith-Guzmán 2015a, 2015b; Soren et al. 1995; Tayles 1996), and they were used in our study to aid in sample selection. (More recently, Smith-Guzmán (2015b) suggests including *cribra femora*, *cribra humeri,* and linear enamel hypoplasias as skeletal indicators of malaria, though this connection has yet to be proven.) Anaemia has a twofold relationship with malaria; it is not only closely associated with the *Plasmodium* parasites, which cause ‘malarial anaemia’ or haemolytic anaemia, but it is also believed to confer a degree of acquired immunity or resistance through inherited blood disorders, such as thalassemia and sickle-cell anaemia (Angel 1966; De Sanctis et al. 2017; Haldane 1949). Thus, conclusive identification of malaria in macroscopic palaeopathological analyses is still not possible and hence justifies the necessity of applying destructive biomolecular approaches, such as immunological and genomic assay in order to investigate malaria in the past.

#### RDTs and malaria

The first immunochromatographic test on ancient human remains was carried out by Miller et al. in 1994 using a rapid manual ParaSight *F*-test and detected malaria antigens in Egyptian mummies dating from 3200 to 1304 BC (Miller et al. 1994). While these early RDTs have since been proven to likely overestimate the rate of *P*. *falciparum* in clinical samples from patients who have rheumatoid factor (Bartoloni et al. 1998; Lafferl et al. 1997; Moody and Chiodini 2002; Wongsrichanalai et al. 2007), the development of a new generation of tests, in which the Immunoglobulin G (IgG) used in the earlier generation has been replaced with Immunoglobulin M (IgM), considerably reduces this cross-reactivity and increases the precision of the identification of malaria proteins (Grobusch et al. 1999; Mishra et al. 1999; Wongsrichanalai et al. 2007). More recently, immunological evidence of malaria infection was found in the mummified muscle tissue of an Egyptian child from the early Dynastic period (Bianucci et al. 2008), in bone samples of four members of the Renaissance Medici Family in Italy (Fornaciari et al. 2010), and in 84 skeletons from the Giza Plateau (Al-Khafif et al. 2018). Fornaciari et al. (2010) used two different types of double-antibody tests with a sterile physiological saline solution for antigens extraction, which allows for the discrimination of *P*. *falciparum* from the other three *Plasmodia* species, while Al-Khafif et al. (2018) have relied solely on a single double-antibody test. Further discussion on this method is provided in Loufouma Mbouaka et al. (2020), and as noted in Nerlich et al. (2008), there are some questions about the specificity of these tests and false-positive results.

#### Ancient DNA and malaria

Ancient malaria DNA was first identified in a single infant skeleton using the polymerase chain reaction (PCR) method, in a sample from Lugnano, Italy (Sallares and Gomzi 2001). The authors provided an excellent overview of the PCR method and the pitfalls associated with it, as well as exploring other methods of identifying malaria in the past. They used primers targeting the plasmodial 18S rRNA genes to identify a likely active malarial infection at the time of death. Sallares and Gomzi are quite conservative about the ramifications of their results, in which they conclusively confirm the presence of *P*. *falciparum* after analysis of the data obtained after PCR and sequencing of the DNA from their samples (Sallares and Gomzi 2001: 203). Since then, there have been several published papers in which authors have improved this method and identified malaria from mummified tissue and in the skeletal material of ancient human remains, along with beginning to establish the evolutionary history and path of the pathogen (Nerlich et al. 2008; Hawass et al. 2010; Marciniak et al. 2016; de Dios et al. 2019; van Dorp et al. 2020). Marciniak et al. (2016) have presented the new gold standard for identifying malaria and its phylogeny, through the capture-enrichment and sequencing of the mitochondrial DNA of the *Plasmodium* parasite which they extracted and identified from Roman material dating from the first and second century CE. The mitochondrial DNA sequences generated during this study provided clear evidence for the presence of malaria in two out of fifty-eight individuals. Further to this, Gelabert et al. (2016) used an extended capture approach to recover a partial genome of eradicated strands of European *P*. *vivax* and *P*. *falciparum* on three different slides containing blood film of Spanish people infected with malaria from the mid-twentieth century. From this analysis, they were able to interpret some of the evolutionary history of malaria in Spain and found similarities between these strains and the American and Indian haplotypes. Recently, using the same slides and methodology, van Dorp et al. (2020) expanded Gelabert et al.’s work and were able to re-construct the complete genome of an ancient European strand of *P*. *vivax*, and further, they reported the presence of some malaria resistant genes in the complete genome.

As it is a significant source of genetic information, as presented above, mitochondrial DNA capture and enrichment of autosomal DNA have become an ideal tool for the investigation and identification of diseases affecting past populations, such as malaria. Thereby, retrieved data can be used to authenticate the results (Warinner et al. 2017) and they provide the means to determine the phylogeny of the *Plasmodium* pathogen. As the most specific and detailed analysis available to identify malaria in the past, aDNA analyses will be compared alongside immunological results and palaeopathological observations to allow for discussion of the positives and negatives of each method.

## Material and methods

### Study samples

**Fig. 1:**
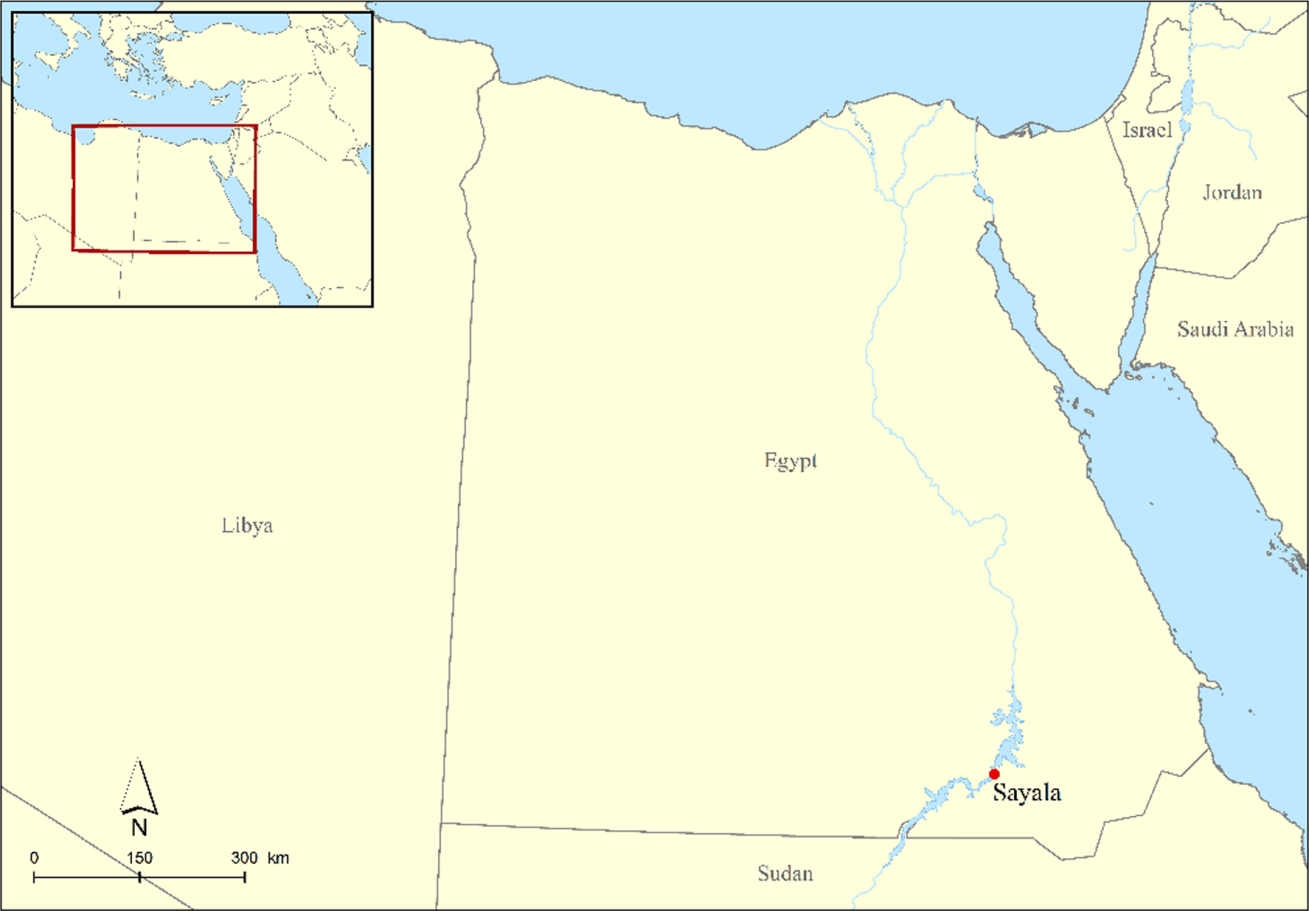
Map location of Sayala, Egypt (C. Kurtze, ÖAI @ ÖAW)

**Fig. 2:**
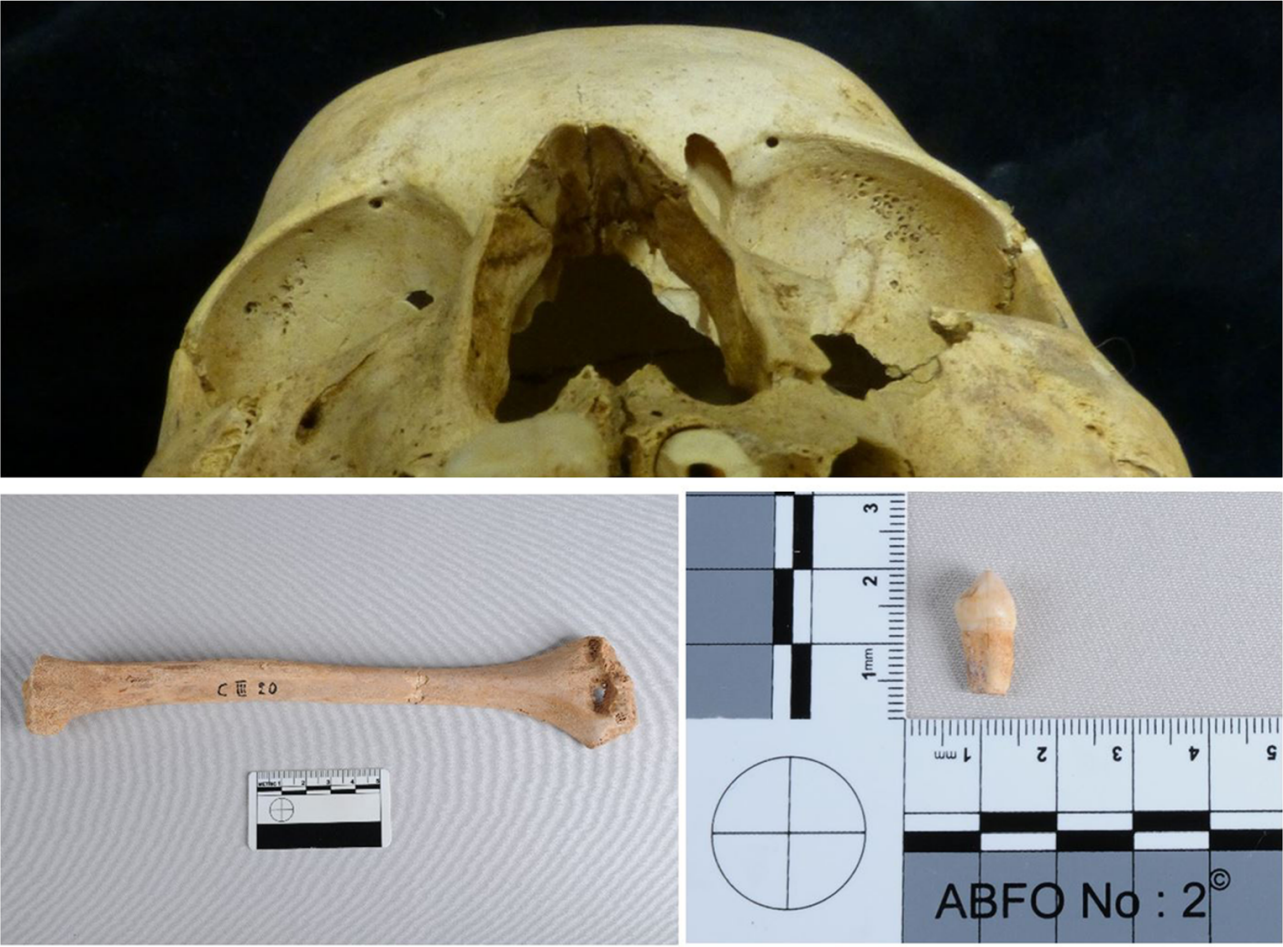
Top image: an example of cribra *orbitalia* (stage 1) (EURAC 2235, 2236 — CIII/20) (photo: M. Gamble, with permission from the NHM, Vienna). Bottom images: an example of a bone and a tooth from Sayala skeletal material and from the same individual (EURAC 2235, 2236 — CIII/20) (photo: A. Loufouma Mbouaka, with permission of the NHM, Vienna)

Ten individuals from the Nubian site of Sayala in Egypt were selected for this study based on a prior macroscopic palaeopathological analysis by E. Strouhal (unpublished report). A tooth and a bone were selected from each individual who was observed to have *cribra orbitalia* (CO) (Table 1; Fig. 2). Sayala is located roughly 120 km south of Aswan (Fig. 1) and was excavated between 1961 and 1965 by the Austrian mission under the Nubian Safeguarding action by UNESCO (Bietak and Jungwirth 1966). The site included up to ten areas excavated as separate cemeteries/burial complexes from various periods (2nd millennium BC, third to sixth centuries AD and eighth to tenth centuries AD), from which 650 individuals were excavated and transported to the Natural History Museum of Vienna, where they are currently stored (Strouhal 1992 and references within). All material examined here derives from cemeteries CI, CII, CIII, and N which date to the third to sixth centuries AD, at which time Sayala was situated near the border of the Roman/Byzantine and Meroitic Empires (Bietak and Jungwirth 1966; Strouhal and Jungwirth 1971, 1979, 1980). Cemeteries CI, CII, and CIII are considered to be roughly contemporaneous (along with cemetery N) and of similar construction, composed of large oval stone mounds which are burial complexes that grew concentrically outwards at each site on the east bank of the Nile (Strouhal and Jungwirth 1971: 12). Cemetery N is slightly different; it is located across the river from cemeteries CI–III and consists of simple pit graves, with a large portion of the population composed of non-adults, yet it dates to roughly the same time as cemeteries C I-III (Strouhal and Jungwirth 1980: 61). The human remains analysis has been summarised by Strouhal and Jungwirth in several publications, including preliminary palaeopathological observations (1971, 1977, 1980, 1982) and an early palaeogenetic study (1979) using non-metric traits and anthropometry (note that these are summaries with a focus on specific aspects of the analysis, while Strouhal’s unpublished report provides in-depth observations by individual, aiding in sample selection).

**Table 1:** List of samples used, including grave and individual, sample number, age, sex, and the bone which was sampled. Note: L = left; R = right; d = deciduous; max = maxillary; man = mandibular; M = molar; PM = premolar; C = canine; I = incisor. * = a tooth with an open root apex

Cem/Gr/Ind	Age (years)	Sex (from aDNA)	Tooth sampled	Bone sampled	EURAC ID
CIII/8/2	13–15	Possible male	L max PM2		2229
				L humerus	2230
CIII/59	12–13	Female	R man PM1*		2231
				L femur	2232
CIII/29	20–27	Possible female	L man M2		2233
				R humerus	2234
CIII/20	12–13	Possible male	R man PM1*		2235
				L humerus	2236
CIII/60	10–12	Possible male	R man I2		2237
				L tibia	2238
CII/139	23–25	Female (osteological ID)	L max I1		2239
				5th lumbar vertebra	2240
CI/47/1	13–16	Possible female	L man PM2*		2241
				L tibia	2242
CI/54/1	12–14	Undetermined	R man PM1*		2243
				L tibia	2244
CI/20	8–9	Possible male	dL max C*		2245
				L humerus	2246
N/12	9–10	Undetermined	dR man M1		2247
				L tibia	2248

The individuals from the graves are recorded here with the cemetery identifier, the grave number, and if there was more than one individual in the grave, then the individual number is also provided (Cem/Gr/Ind). Each individual has two EURAC laboratory identifiers — a number for the tooth sample and a number for the bone sample (Fig. 2).

### Palaeopathological re-analysis

The ten individuals from Sayala were all re-examined macroscopically at the Natural History Museum, Vienna. Standard methods of assessment were applied to assess for age, sex, and any pathological changes (Brickley and McKinley 2004; Buikstra 2019; Buikstra and Ubelaker 1994; Mitchell and Brickley 2017; Schaefer et al. 2009; White and Folkens 2005 — and references included within each). In particular, grades were applied to determine the state and extent of CO in each individual. The grades used for this study are illustrated by Rivera and Mirazón Lahr (2017) who based their scale on that of Stuart-Macadam (1991) and Buikstra and Ubelaker (1994). This system is based on ascending grades: Grade 1 shows isolated pores on the roof of one of both orbits; grade 2 shows isolated pores with some coalescing to give a ‘net-like’ appearance; grade 3 shows coalesced pores which are merged and look like ‘netting’; grade 4 shows enlarged or coalesced pores which have a thick, honeycomb appearance (with no protrusions); and grade 5 shows protruding trabecular bone from the outer table with a honeycomb appearance (Rivera and Mirazón Lahr 2017: 78–79).

### Antigen extraction and analysis

The immunoassay analysis was performed at the Institute of Specific Prophylaxis and Tropical Medicine, Medical University of Vienna. To avoid any contamination by modern genetic material, ancient DNA extraction and analysis were performed before antigen extraction. The antigen extraction was performed according to the published protocols of Bianucci et al. (2008) and Fornaciari et al. (2010), and only bone material was included in the study, since teeth do not provide enough material for both ancient DNA and antigen analyses. Two types of rapid diagnostic tests (RDTs) were applied for this study: (1) malaria card test Pv/Pf, DiaSys (DiaSys Diagnostic System GmbH, Holzheim, Germany) which detects the HRP2 of *P*. *falciparum* and pLDH specific to *P. vivax*; (2) malaria card test Pan/Pf, DiaSys (DiaSys Diagnostic System GmbH, Holzheim, Germany) for the detection of the HRP2 antigen of *P*. *falciparum* and pLDH for a pan-malarial antigen. For further details regarding the methods employed here, see Loufouma Mbouaka et al. (2020) and the online supplementary notes.

### Ancient DNA analysis

The genomic analysis was carried out at the ancient DNA laboratory of the Eurac Research — Institute for Mummies Studies, based in Bolzano, Italy. All 20 samples from the ten individuals were documented with photographs and then cleaned with hydrogen peroxide and subjected to a UV light. In order to collect the necessary skeletal powder: (1) The tooth was transversally split with a clean and disinfected Dremel diamond blade and the inner part, composed of pulp and dentine, was collected with a driller and weighed, and (2) a hole was created with a single-use drill bit in the bone at a point where the dense medullary bone was expected, and the interior surface of the cortical bone was collected and weighed. Approximately 60 mg of tooth powder and 230 mg of bone powder were taken for each respective sample and subjected to a silica-based DNA extraction (Rohland et al. 2010; Damgaard et al. 2015). A DNA extraction blank control was converted to an Illumina library and also subjected to shotgun sequencing. The library preparation was performed according to the published protocol of Meyer and Kircher (2010). Thereby, a unique P5 and P7 index combination was added to each library. A library preparation control (PCR-grade water) was included for every five samples. The libraries were first subjected to a paired-end shotgun sequencing approach, and subsequently, a capture-sequencing approach targeting the *Plasmodium* mitochondrial genome, as described by Marciniak et al. (2016), was applied to selected samples that had first indications for the presence of *Plasmodium* DNA.

All 20 barcoded samples were first subjected to shotgun sequencing on an Illumina HiSeq 2500 (2 × 101 cycles) platform. Paired Illumina reads were quality-checked and processed (adapter removal and read merging) using the SeqPrep tool v1.2 (https://github.com/jstjohn/SeqPrep-SeqPrep-fforward-read-fastqfile-rreverse-read-fastqfile-1forward-read-output-fastqfile-2reverse-read-output-fastqfile-L15–A10“AGATCGGAAGAGCACACGTCTGAA”-B“AGATCGGAAGAGCGTCGTGTAGGG”–smerged-fastq-file). Pre-processed shotgun reads were first aligned to the human full genome (build Hg19 — Rosenbloom et al. 2015) and the human mtDNA reference genome rCRS (Andrews et al. 1999) using bowtie2 (v1.2.1.1) and the parameter ‘end-to-end’ (Langmead and Salzberg 2012). To deduplicate the mapped reads, we used the DeDup tool v0.12.8 (https://github.com/apeltzer/DeDup). The minimum mapping and base quality were both 30. The resulting bam files were used to check for characteristic aDNA nucleotide misincorporation frequency patterns using mapDamage2 v.2.0.9 (Jónsson et al. 2013). The sex of the individual was assigned using a maximum likelihood method, based on the karyotype frequency of the mapped human X and Y chromosomal reads (Skoglund et al. 2013). In one sample with enough human mitochondrial reads (EURAC 2240 — CII/139), the rate of human contamination was estimated using Schmutzi v1.5.1 (Renaud et al. 2015). Variants in the mitochondrial genome of sample EURAC 2240 were called using SAMtools mpileup and bcftoools (Li et al. 2009) with stringent filtering options (quality > 30). Visual inspection of the called variants identified only less than 1% low-frequency variants that could be indicative for contamination. The haplogroup was identified by submitting the variant calling file to the HaploGrep website (Weissensteiner et al. 2016). First, we assessed a general taxonomic profile of the sequencing reads using DIAMOND blastx search (Buchfink et al. 2015) against the RefSeq non-redundant protein database (nr). The DIAMOND tables were converted to rma6 (blast2rma tool) format (--minPercentIdentity 97), imported into MEGAN6 software (Huson et al. 2016), and subsequently visualised using the Krona tool (Ondov et al. 2011). Next, the shotgun reads were aligned against the full genome assemblies of *Plasmodium falciparum* (GCA_000002765) and *Plasmodium vivax* (GCA_900093554.1, excluding unplaced sequences) and against the mitochondrial genomes of both organisms (LR605957.1, LT635627.1) using BWA (Li and Durbin, 2010) with the parameters described by Marciniak and colleagues (-n 0.01 -o 2 -l 16500) (Marciniak et al. 2016). AT-rich repetitive regions were not excluded from the references. Thereby, obtained results with minimum mapping quality 20 and 30 were compared to each other. Subsequently, a sequence similarity search of all mapped reads against the full *Plasmodium* assemblies using blastn (Altschul et al. 1990) and the complete NCBI-nt database (N.R. Coordinators 2017) was performed. Blast results were taxonomically assigned using MEGAN6 and the LCA (Lowest Common Ancestor) algorithm (Huson et al. 2016). To identify false-positive assigned reads, we manually subjected once more the LCA-assigned *Plasmodium* reads to sequence similarity search using blastn against the NCBI nt database. Thereby, we only considered reads being unambigiously assigned to *Plasmodium*, when this assignment appeared as top blast hit. Selected samples (EURAC 2229 (CIII/8/2), 2230 (CIII/8/2), 2239 (CII/139), 2240 (CII/139)) were further subjected to a capture-enrichment assay targeting *Plasmodium* mitochondrial genomes. The capture in-solution bait assay was designed according to Marciniak et al. (2016) by including the mitochondrial sequences of six *Plasmodium* species (*P*. *falciparum*, NC_002375; *P*. *vivax*, NC_007243; *P*. *malariae*, AB_354570; *P*. *oval*e, AB_354571; *P*. *knowlesi*, NC_007232; and *P*. *cynomolgi*, AB_434919) and excluding parts of the COXIII gene (*P*. *falciparum*, positions 1522–1557) and positions within the intergenic and rRNA regions (*P*. *falciparum*, positions 5707–5754) that previously displayed high rates of unspecific bindings (for additional details to the baits design, please refer to Marciniak et al. 2016). In-solution enrichment was performed according to the manufacturer’s protocol (Custom myBaits-1 16 Rxn Kit, Arbor Biosciences, Ann Arbor, MI) with minor modifications (hybridization time 48h). Two samples, EURAC 2247, 2248-N/12, were included as a negative control into the capture assay since they did not show any signs for the presence of *Plasmodium* DNA in the initial shotgun screening. Captured Illumina libraries were sequenced on a HiSeqX platform (2 × 151 cycles). Thereby, retrieved sequence reads were subjected to the same pre-processing and analysis pipelines as described before for the shotgun sequence reads. Mitochondrial capture results were compared to the captured sequence data of two *Plasmodium* positive samples (LG20 and LV13) from Imperial period Italy published by Marciniak and colleagues (SRR4425648, SRR4425649) (Marciniak et al. 2016). For details to the Illumina datasets and all mapping results, please refer to the Supplementary table S1. Data are available from the European Nucleotide Archive under accession no. PRJEB43969.

## Results

### Palaeopathological re-analysis

**Fig. 3:**
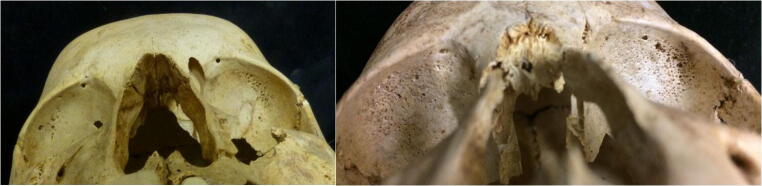
Examples of the CO observed on the skeletal material from Sayala. Both the right and left images are from the inferior-anterior aspect with the superior aspect of the cranium to the top of the image. Left photo: superior aspect of orbits from cemetery III, grave 20 (EURAC 2235, 2236) with stage 1 CO. Right photo: superior aspect of orbits from cemetery I, grave 20 (EURAC 2245, 2246) with stage 4 CO (photo: M.Gamble, with permission of the NHM, Vienna)

While re-analysis of the entire corpus of skeletal material from the four cemeteries explored in this paper was not possible, the 10 individuals, from whom biomolecular samples were taken, were re-examined to see if there was anything new to contribute to the issue of identifying malaria in the past. A brief summary of the palaeopathological results is presented in Table 2, but it must be remembered that these individuals were selected because they were observed by Strouhal to have CO or PH (unpublished report). Therefore, in order to discuss the severity of CO, grades were assigned. Only one individual displayed possible healed or healing porotic hyperostosis of the cranial vault (CIII/8/2 — EURAC 2229, 2230). There was a spectrum of severity of CO observed (Fig. 3; Supplementary Figures SA-SJ) across all individuals, from mild to more severe porosity, along with several other pathologies.

**Table 2:** Brief summary of the key pathological symptoms observed (note: 1 = present; 0 = not present; CBA= cannot be assessed; Cem = cemetery; Gr = grave; Ind = individual; CO = *cribra orbitalia* (see above for grade descriptions); PH = porotic hyperostosis; LEH = linear enamel hypoplasia; Dental path = caries, calculus, and/or alveolar porosity/recession). Note that ^1^ represents the osteological sex and ^2^ represents the molecular sex obtained after DNA analysis

EURAC ID	Cem/Gr/Ind	Age	Sex^1^	Sex^2^	CO grade	PH	LEH	Dental path	Cribra femora	Cribra humeri
2229 2230	CIII/8/2	13–15 y	CBA	M	3	Healed?	1	0	Possibly	0
2231 2232	CIII/59	12–13 y	CBA	F	2	0	0	0	Possibly	0
2233 2234	CIII/29	20–27 y	F	F	4	0	0	1	0	0
2235 2236	CIII/20	12–13 y	CBA	F	1	0	0	1	0	0
2237 2238	CIII/60	10–12 y	CBA	M	2	0	1	0	Possibly	Possibly
2239 2240	CII/139	23–25 y	F	F	2	0	CBA	1	Possibly	0
2241 2242	CI/47/1	13–16 y	CBA	F	1	0	0	1	Possibly	0
2243 2244	CI/54/1	12–14 y	CBA	F	2	0	1	0	Possibly	Possibly
2245 2246	CI/20	8–9 y	CBA	F	4	0	0	1	Possibly	0
2247 2248	N/12	9–10 y	CBA	M	3-4	0	0	0	Possibly	Possibly

**Fig. 4:**
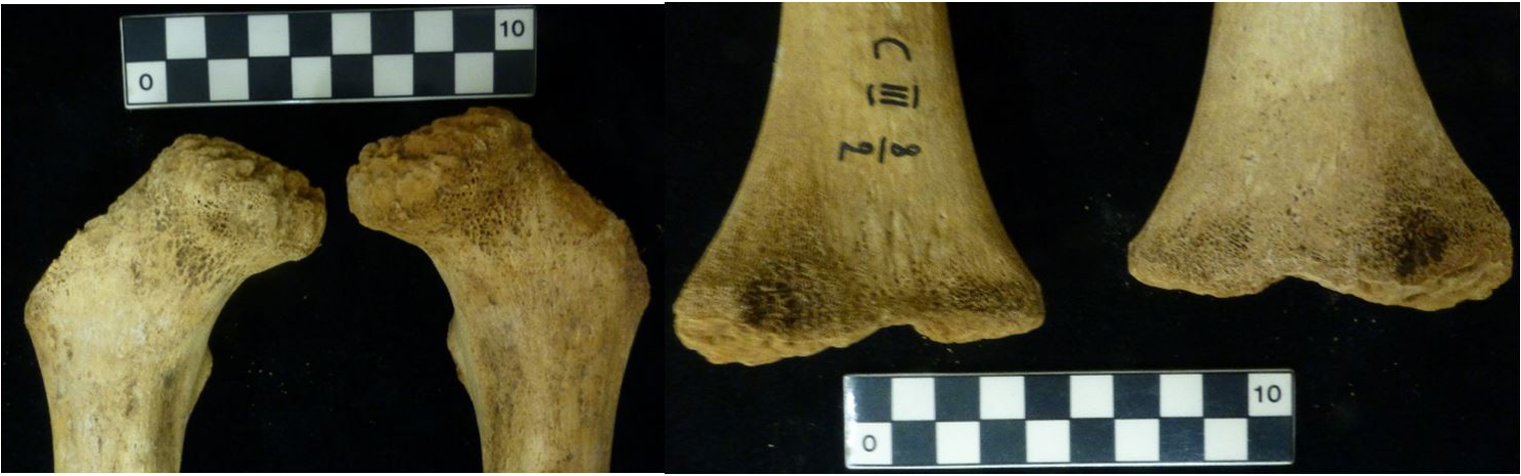
Example of the porosity observed at the femoral necks (left) and popliteal surface/distal metaphyses (right) of the femora from cemetery III, grave 8, individual 2 (EURAC 2229, 2230). Left photo: proximal end of the right and left femora, medial-anterior view. Right photo: distal end of the left and right femora, posterior view (photo: M.Gamble, with permission of the NHM, Vienna)

All the non-adult individuals examined displayed significant levels of porosity and woven bone growth at the metaphyses of the large long bones (i.e. the humeri, the femora, and the tibiae) (Fig. 4). It is difficult to attribute this observation to *cribra femora* or *cribra humeri*, as the identification of these specific pathologies is problematic amongst non-adults as natural bone growth and development have a similar appearance in young individuals (Lewis 2018: 193–195). Therefore, it was not possible to diagnose a cribrotic disease in these individuals, and thus, it cannot be conclusively associated with a response to an anaemic condition at this level of analysis. The results of the macroscopic analysis of skeletal changes in regard to the presence of malaria are inconclusive.

### Immunoassay using rapid diagnostic tests

The immunological assessment was performed only with the bone samples from the ten individuals, since there was not enough material remaining after the DNA analysis to perform immunoassays with tooth samples (all previously reported in Loufouma Mbouaka et al. 2020). After antigen extraction and analysis with the two different rapid diagnostic tests (RDTs), both tests detected four positive samples of malaria: EURAC 2230, 2240, 2242, and 2244 (CIII/8/2, CII/139, CI/47/1, CI/54/1, respectively), while 2234 (CIII/29) showed a positive with only one type of test (QDx Malaria card DiaSys Pan/Pf test) (Table 3; Figure SV). Three bone samples from skeletal human remains dating to the early nineteenth century excavated at Aspern Seestadt, a district located in the north-east of Vienna, were used as a control and were all negative for *Plasmodium* antigens, as hoped. To summarise, 40% (*n*=4) of the samples presented positive results for *Plasmodium falciparum* and/or *Plasmodium* vivax, and/or the pan-malaria, with a further sample testing positive for the pan malaria antigen only.

**Table 3:** Results obtained with QDx Malaria card test DiaSys Pv/Pf and QDx Malaria card test Diasys Pan/Pf

EURAC ID	Cemetery/grave/individual	QDx Malaria card test DiaSys			
		Pv/Pf		Pan/Pf	
		Pv	Pf	Pan	Pf
2230	CIII/8/2	+	+	+	**+**
2232	CIII/59	-	-	-	-
2234	CIII/29	-	-	+	**-**
2236	CIII/20	-	-	-	-
2238	CIII/60	-	-	-	-
2240	CII/139	+	+	+	**+**
2242	CI/47/1	+	+	+	**+**
2244	CI/54/1	+	+	+	**+**
2246	CI/20	-	-	-	-
2248	N/12	-	-	-	-
Aspern Seestadt 2012 (SA)		-	-	-	-
Aspern Seestadt V33 (V33)		-	-	-	-
Aspern Seestadt Unknown (SU)		-	-	-	-

### Ancient DNA analysis

To assess the quantity and quality of the extracted DNA, both shotgun sequencing and capture sequencing were performed and the sequence reads were mapped against the human genome and subjected to a first taxonomic overview using DIAMOND against the nr database. The majority of reads in the samples are assigned to the bacteria. Less than 5% of the reads were eukaryotic with up to 95% fungal reads (Figure SWA and B). Overall, the human endogenous DNA content was very low and ranged between 0.0005 and 0.083% (Table 4; Supplementary Table S1). Only one individual contained sufficient human mitochondrial reads to reconstruct the full mitochondrial genome with a mean coverage of 15.8 (EURAC 2240-cemetery II, grave 139). Deamination patterns of approximately 18% at the 5′ and 3′ ends of the human mitochondrial reads were observed, typical for ancient DNA damage (Supplementary Figure SXA). The estimation of approximately 2% contamination of the uniparental marker obtained with the software Schmutzi further supports the authenticity of the detected ancient human DNA in this individual (Supplementary Table S1). Finally, the individual’s mtDNA haplogroup could be successfully assigned to the sub-haplogroup J1c of the macrohaplogroup J (Supplementary Figure SXB). The mtDNA macrohaplogroup J has been previously detected in ancient mummified human remains from the archaeological site Abusir-el Meleq in Egypt (Schuenemann et al. 2017).

**Table 4:** Shotgun and capture (in bold) sequencing output and mapping data against human and *Plasmodium* reference genomes. Listed are all individuals that show first indications for the presence of *Plasmodium* DNA. For more details to the results of all analysed specimen, please refer to the Supplementary Table S1

EURAC ID	Individual	Sample	% human endogenous DNA	Genetic sex	No. of human mtDNA reads	Haplo-group human mtDNA	No. of reads mapped to the *P*. *falciparum/vivax* full assembly, Dedup, MQ> 20	*Plasmodia* spp. reads after blastN vs. NCBI nt and LCA assignment and re-evaluation of the Blast hit (*P*. *falciparum*/*vivax*)	No. of reads mapped to the *P*. *falciparum/vivax* mtDNA genome, Dedup, MQ > 30
2229	Cemetery CIII, grave 8, individual 2	Tooth	0.0466	XY	25		422/2119	1/0	19/18
**2229**		**Tooth**	**0.0834**	**XY**	**94**		**2141/6335**	**3/0**	**164/224**
2230		Bone	0.0023	Not assigned	2		251/1087	0/0	9/9
**2230**		**Bone**	**0.0062**	**Not assigned**	**5**		**1140/3233**	**0/0**	**137/176**
2239	Cemetery CII, grave 139	Tooth	0.0050	Consistent with XX	0		34/515	0/0	0/0
**2239**		**Tooth**	**0.0083**	**Not assigned**	**0**		**2/6**	**0/0**	**0/0**
2240		Bone	0.0034	Consistent with XX	0		114/520	0/0	7/7
**2240**		**Bone**	**0.0225**	**Consistent with XX but Not XY**	**2050**	**J1c**	**437/2475**	**1/1**	**116/168**
2241	Cemetery CI, grave 47, individual 1	Tooth	0.0003	Consistent with XX	0		39/716	0/0	2/2
2242		Bone	0.0015	Not assigned	0		60/2049	0/1	5/5
2243	Cemetery CI, grave 54, individual 1	Tooth	0.0041	Not assigned	0		38/541	0/0	1/1
2244		Bone	0.0005	Consistent with XX	0		118/2381	0/1	8/8
2245	Cemetery CI, grave 20	Tooth	0.0027	Consistent with XX	0		89/1362	0/0	10/10
2246		Bone	0.0012	Consistent with XX	1		106/1917	1/0	8/8

The general taxonomic overview revealed by the DIAMOND analysis already indicated the presence of *Plasmodium* DNA in selected samples (figure SWA). We further investigated this observation by mapping both shotgun and capture sequencing data against the genomes of *P*. *falciparum* and *P*. *vivax* and by subjecting the mapped reads to further taxonomic assignment. The number of reads that mapped against the genomes of *P*. *falciparum* and *P*. *vivax* varied substantially between the samples and ranged from 2 to 6335 reads (Table 4; Supplementary Table S1). Approximately three to ten times more reads were mapped throughout all samples to the *P*. *vivax* genome. The current draft version of the *P*. *vivax* assembly, however, is known to contain human and bacterial background contamination which could explain the higher proportion of mapped reads (Lu and Salzberg 2018). Finally, to obtain a taxonomic overview of the mapped reads and to identify the most prevalent taxonomic groups in the samples, a sequence similarity search using blastn was performed, with default parameters and the complete NCBI-nt database as the reference database. Blastn results were taxonomically assigned using the LCA algorithm in MEGAN6. Thereby, most mapped reads were taxonomically assigned to the human and various bacterial and fungal species. Five individuals showed indications for the presence of *Plasmodium* DNA which resulted after manual re-evaluation of LCA assigned reads using blastN against the NCBI nt database and keeping the top hits only in eight taxonomically assigned *Plasmodium* reads (Table 4; Figure SY). Due to this very low number of reads neither any further assessment of authentication criteria for the detected DNA (e.g. damage pattern, even coverage of the genome) nor any species assignment was possible. Therefore, it was decided to apply a *Plasmodium* specific mitochondrial genome capture, as described by Marciniak et al. (2016), on two individuals which showed indications for the presence of *Plasmodium* DNA already in the shotgun datasets (EURAC 2229, 2230 — CIII/8/2 and EURAC 2239, 2240 — CII/139). In addition, the capture assay of two samples (EURAC 2247, 2248-N/12) that contained no *Plasmodium* DNA in the shotgun datasets were included in as negative control, and for the subsequent data analysis, two individuals from the study of Marciniak et al. (2016) were added in as positive control that showed a positive *Plasmodium* mitochondrial DNA enrichment effect.

In general, for all samples including the negative controls, the number of mapped reads to the *Plasmodium* mtDNA genomes increased after capture (Table 4; Supplementary Table S1). A closer look on the read distribution however revealed that most captured reads accumulate at two regions in the mitochondrial genome, the small and the large subunit ribosomal RNA fragment E (Supplementary Figure SZ). In contrast, the reads of the two positive control samples from the study of Marciniak et al. (2016) show an even distribution of reads. The occurrence of read stacks at the 3′ termini of the COXIII gene and within the intergenic and rRNA regions has been already observed in this previous publication. Therefore, these regions were excluded in the capture design. Still, however, these regions were preferentially present in our captured datasets. One possible explanation could be the increased GC content in this regions that could facilitate the capture of unspecific homologous non-*Plasmodium* DNA (Figure SZ). Overall, the retrieved data allowed no further authentication (e.g. damage pattern analysis) of the reads or *Plasmodium* species assignment.

### Comparative analysis

Both the immunological assay and the aDNA results reveal consistent indications of the presence of *Plasmodium* antibodies and DNA in four out of the ten tested individuals (Table 5). While samples 2234 (CIII/29) and 2246 (CI/20) only obtained a positive result with one of the analyses, immunological and genetic, respectively, there was similarity between the rest of the results of the biomolecular analyses. This means that four of ten of the individuals tested provided consistent positive indications for the presence of *Plasmodium*, both immunologically and genetically. The only tooth to provide a positive result for the presence of *Plasmodium* DNA was from sample 2229 and had a correlating result from the bone from that individual (2230 — CIII/8/2). The four individuals who tested positive for the presence of malaria did not display any consistent pathological lesions which set them apart from the individuals who were negative for malaria. The following section will provide a discussion of the results and some of the implications of this study for the detection of malaria in ancient human remains.

**Table 5:** Comparative results of the biomolecular analyses and pathological lesions of the individuals who tested positive for the presence of either *Plasmodium* antibodies or aDNA. *Sample 2229 was the only positive tooth sample from the aDNA analysis and was not tested using the RDTs (see above for CO grade descriptions; for LEH results — 1 = present, 0 = not present, CBA = cannot be assessed)

EURAC ID		Genetic sex		RDT				DNA	CO grade	LEH
	Cem/Gr/Ind		Sample type	Pv/Pf		Pan/Pf				
				Pv	Pf	Pan	Pf			
2229*, 2230	CIII/8/2	Male	LMaxPM2*, L humerus	+	+	+	+	+	3	1
2234	CIII/29	Possible female	R humerus	-	-	+	**-**	-	4	0
2240	CII/139	Probable female	5th lumbar vertebra	**+**	+	+	+	+	2	CBA
2242	CI/47/1	Possible female	L tibia	+	+	+	+	+	1	0
2244	CI/54/1	Possible female	L tibia	+	+	+	+	**+**	2	1
2246	CI/20	Probable female	L humerus	-	-	-	-	+	4	0

## Discussion

This research has provided the first comparative study between two widely used biomolecular techniques and skeletal morphological changes in the investigation and identification of malaria in a past population. Previous research on the identification of malaria in archaeologically derived human remains has used only one approach: immunoassay (i.e. Bianucci et al. 2008; Fornaciari et al. 2010), or genomic identification (i.e. Marciniak et al. 2016), or macroscopic palaeopathology and interpretation (i.e. Angel 1966, 1978; Gowland and Garnsey 2010; Gowland and Western 2012; Smith-Guzmán 2015a). Since each method seeks different aspects of the disease, antibody production and presence, or the presence of the *Plasmodium* parasite DNA, or evidence of haemolytic anaemia resulting in porosity in the bones, it is particularly useful that the results show some correlation. This section will present our discussion on the results obtained with each method, with particular focus on the limits of each, including biomolecule survival, test specificity and sensitivity, and sample selection. We conclude that at this stage of research into malaria in the past, it is important to apply multiple techniques to establish the presence of malaria in ancient human remains and ensure authentication of the results for conclusive statements to be made.

### Macroscopic palaeopathological assessment

It must be remembered that for the skeleton to be involved in the symptoms of a disease, the disease must be chronic, and the individual must have survived long enough for osseous involvement (Wood et al. 1992). Therefore, these individuals would have had to survive the infection or multiple infections for some time, which means that the correlation between the immunological/genetic results and the macroscopic results may be very weak, as a higher parasitaemia load is expected to be more likely to be detected by the immunoassays and genetic analysis, as well as being more symptomatic for the individual which could possibly also result in earlier death (this relates to the Osteological Paradox — Wood et al. 1992; Wright and Yoder 2003; DeWitte and Stojanowski 2015; while parasite density and morbidity has been explored by Ali et al. 2008). Clinically, it can be difficult to determine the effect of the parasite density, as many individuals in endemic malarial areas are asymptomatic (not displaying the typical cyclical febrile attacks associated with malaria) and thus are not included as often in studies, which rely on those seeking medical attention for their symptoms (Galatas et al. 2016). There is research exploring the impact of asymptomatic *Plasmodium* infection on society (part of the malarial burden); however, asymptomatic individuals pose a conundrum for our analysis, as typically they carry a lower parasite density, yet may be more likely to suffer from anaemia (due to the protection it confers for malaria), and thus, the skeletal response would reflect the anaemia, while the biomolecular results may not support a malaria diagnosis in archaeological populations in endemic areas (Galatas et al. 2016). All the non-adults within this study show high levels of vascularization of the vertebrae and at the metaphyses with porosity and, in the case of the metaphyses, woven bone growth. Since there is no visible difference between those with *Plasmodium* positive biomolecular/genetic tests, and those who are negative, this observation cannot be directly linked to a specific disease, and most likely represent the ubiquitous childhood growth and development range of changes (Lewis 2018: 196). In the case of haemopoietic diseases, children have more reactive bone marrow than adults, so they can present more severe skeletal changes with relatively smaller stimuli, though it still must occur for some time (Bolton-Maggs and Thomas 2008; Lewis 2018: 193). While there were some indications of possible porosity at the metaphyses of the long bones, our results do not support the use of *cribra femora* and *cribra humeri*, particularly with non-adult individuals, as a conclusive indicator of malaria (as suggested by Smith-Guzmán 2015b — however, this would be best tested with a documented skeletal collection).

There is widespread discussion in palaeopathology about the aetiology of and connection between porotic hyperostosis (PH) and *cribra orbitalia* (CO) (see Brickley 2018 for a synopsis and above). The different underlying causes of these lesions include iron-deficiency anaemia or megaloblastic and haemolytic anaemia or other physiological stresses causing porosity and cortical thinning, such as nutritional deficiencies like scurvy or rickets or localised infection (see i.e. Ortner 2003 and Aufderheide and Rodríguez-Martín 1998 and most recently, O’Donnell et al. 2020 associate CO and PH with respiratory infections). While Strouhal did not include PH explicitly in his analysis, he presents the prevalence of CO across the population he observed (Strouhal unpublished: section 11.5). Overall, he notes that of the 63 infants and children, only 22 had intact orbital roofs, of which 63.6% (*n* = 14) displayed some level of porosity, while only 14.5% of adults with intact orbits (*n* = 227) exhibited porosity (*n* = 33), with females displaying more severe cases and a higher prevalence (18.2% compared to males 11.7%) (Strouhal unpublished: section 11.5). Given the difficulties in attributing PH and CO to a specific aetiology, it is not possible to directly tie this pathology to malaria; rather, they are non-specific indicators of a physiological stress. The presence of CO in all of the individuals who were sampled for biomolecular/genetic analyses in our study highlights the complex nature of identifying the presence of malaria in the past macroscopically, as individuals who provided *Plasmodium* positive biomolecular results display this pathological change, as do those who did provide negative tests.

### Immunological results

The results of the immunological analyses are presented in greater detail in Loufouma Mbouaka et al. (2020), but to summarise, we have included some detail in the Online Supplementary Material, and note here that there are a number of factors which we have determined will significantly impact the results we obtained. These factors include the following: antigen survival, taphonomy, RDT sensitivity, and sample selection. Primarily, there are significant questions regarding the survival of the antigens detected by the RDTs (*Plasmodium falciparum* histidine–rich protein-2 (pfHRP2) which is specific to the P. falciparum species and parasite lactate dehydrogenase (pLDH) which is an example of a pan-/species-specific malaria antigen), and subsequently, the ability of the commercially available rapid diagnostic tests (RDTs) to detect the fragmented or small quantities of these antigens which are likely present in ancient remains. Research seems to suggest that the antigens that the RDTs detect can remain measurable in the body from 5 days to up to 2 months after treatment and can still be detected after death caused by malaria (Berens-Riha et al. 2009; Dalrymple et al. 2018). This, alongside the results of previous research (i.e. Al-Khafif et al. 2018; Fornaciari et al. 2010), implies that the antigens, associated with malaria, do survive within bone material; however, proteomics will help confirm this in the future. The portion of the skeleton selected for analysis also needs to be considered and will be discussed in more detail below as it relates to both aDNA results and immunological results.

Overall, of the ten bone samples from the ten different individuals analysed from the Sayala cemeteries, five produced a positive result for the presence of antigens associated with malaria. Of these five positive results, two were from the humerus, two from the tibia, and one from a lumbar vertebra, suggesting that multiple bones can retain malarial proteins. Since teeth were not tested immunologically, we cannot comment on the efficacy of using teeth in this type of testing. The sex of each individual was determined through genetic analysis, resulting in four females and one male with a positive immunological test (see Table 5 and discussion below in the ‘Malaria in Sayala in the Roman and Byzantine periods’ section).

### Ancient DNA results

The aDNA results present a challenge as the initial shotgun sequencing could not be authenticated with the mtDNA capture. Our initial results from the shotgun sequencing indicated that five bones and one tooth potentially retained *Plasmodium* DNA, in five different individuals (see Table 5). However, the very low number of reads allowed no further authentication of the detected DNA (e.g. damage pattern, even coverage of the genome with reads) and no *Plasmodium* species assignment. The subsequently performed capture assay targeting the mitochondrial *Plasmodium* genome preferentially enriched two regions in the genome and did not yield further *Plasmodium* DNA. Therefore, the current DNA results display only a first indication for the presence of *Plasmodium* DNA which could not be supported by rigorous authenticity and contamination tests. ‘Unexpectedly, most individuals which tested positive for malaria DNA also showed a positive immunoassay result. This interesting observation in detecting *Plasmodium* biomolecules in the same individuals awaits further investigation. Currently, however, we have to face the limits of detecting ancient DNA in this highly degraded material. DNA becomes highly degraded and damaged after the death of an organism (Kendall et al. 2018: 31). This process accelerates in humid and hot environments (Bollongino et al. 2008). Furthermore, the AT-rich *Plasmodium* genome is possibly more prone to degradation processes since the higher the GC content gets, the more stable the DNA becomes (Yakovchuk et al. 2006). Therefore, we may expect only minute amounts of *Plasmodium* DNA in these samples which are difficult to enrich. Overall, this study confirms the difficulty in retrieving DNA-based *Plasmodium* indications which meet the authentication criteria for ancient DNA in a larger skeletal series (Marciniak et al. 2016). The DNA-based analysis of malaria in the past is currently restricted to the best preserved ancient human remains and needs to be complimented with additional assays targeting other biomolecules such as proteins.

### Preservation of ancient biomolecules

Environmental factors are variable and extremely important for ancient biomolecular preservation. Kendall et al. (2018) have published a helpful synopsis of the different external and intrinsic factors of decomposition and burial which will impact the preservation of the mineralised tissue. In general, the type of the burial, the local environment (i.e. temperature, aridity, and soil composition), manipulation after excavation, and their storage (i.e. Cappellini et al. 2018; Wills et al. 2014) will impact on the preservation of proteins and genetic material. Fluctuating water levels seem to be the most damaging to skeletal material, and it is generally accepted that DNA and proteins do not survive as well in high temperature locations such as the tropics or the Sahara Desert (Bollongino et al. 2008; Hofreiter et al. 2015; Wadsworth et al. 2017). As well, previous studies indicate that freshly excavated bones provide the best results regarding DNA amplification (Pruvost et al. 2007) and protein recovery (Wiechmann et al. 1999). This may have an impact on the Sayala skeletons, which were fairly well-preserved macroscopically, but were excavated in the 1960s. They had subsequently been transported from Egypt to Vienna where they were appropriately curated in the anthropology stores of the Natural History Museum. Protein decomposition or molecular and chemical degradation of DNA may damage the molecular targets and generate false negative or lead to false positive results after cross-reactivity with unspecific targets (Nerlich 2016: 4, Marciniak 2016: 83). Thus, there are a number of external factors which will impact on the preservation of the *Plasmodium* antigens and DNA.

The species of *Plasmodium* which has infected the individual may also have an impact on the results, as those with *P*. *falciparum* not only display more severe symptoms and are the leading cause of death in malaria cases but also tend to have a higher parasitaemia-load which would perhaps allow for better survival of the biomolecules in ancient remains (Rook et al. 2014: 3; Tangpukdee et al. 2012: 325) However, those with *P*. *vivax* would still suffer with cyclical fevers which would have a significant impact on the individual and more widely on the community as part of the malarial burden which relates to the loss of industry and activity due to chronic illness (WHO 2015). There are two issues with understanding the species of *Plasmodium* affecting an ancient community, the first is with the lethality of the parasite which kills before affecting the skeleton, therefore avoiding macroscopic detection and selection for analysis, and secondly, the differential survival of the *Plasmodia* species, which is an unexamined aspect. Therefore, it is possible that different *Plasmodium* species will be more or less detectable based on their quantity within the individual and their survivability over time.

### Test sensitivity and specificity

Common issues which can impact the detection of *Plasmodium* DNA and antigens in a clinical setting, as well as with ancient samples, are the loss of DNA sensitivity over time due to its degradation and the high sensitivity and low specificity of antigen tests (RDTs) (Calarco et al. 2020; Gunawardena and Karunaweera 2015). While there are constant improvements in the detection of ever smaller fragments of aDNA, we are still faced with the limitation of the technology at this time to detect small fragments of *Plasmodium* DNA. This will continue to improve with the enthusiasm which is being shown for working with ever older biological remains (Dabney et al. 2013; Rohland et al. 2018). Unfortunately, the situation is not as clear with the immunological analyses, as these tests, particularly ELISA tests, tend to be highly sensitive, but also highly specific. This specificity may limit the ability of the tests to identify fragments of antigens, which are more likely in older remains. The primary issues with antigen identification are related to specificity and sensitivity, with ELISA tests perhaps too specific, while differing levels of sensitivity across RDTs may impact results. Clinically, the QDx Malaria Card DiaSys tests need only 5 μl of blood sample, from which we can infer that little antigen presence is required for identification in these tests. According to the test kit product instructions of QDx Malaria Card DiaSys test, the test has a sensitivity between 95.5 and 99.8% for 50 parasites/μl (see test kit product instructions of QDx Malaria Pv/Pf performance characteristics). Assuming that the results are not false-positives, it is possible that the antibodies on the QDx Malaria Card DiaSys are able to bind to a smaller fragment of the degraded antigen than other less sensitive tests (Loufouma Mbouaka et al. 2020). Therefore, it would be interesting to know whether QDx Malaria Card DiaSys is using monoclonal antibodies (more specific but less likely to bind to fragments) or polyclonals (which are more likely to bind but also less specific and more likely to cross-react), which unfortunately would require cooperation with the test manufacturers. Therefore, until we can establish the particular antigens which are binding on a positive RDT for malaria, we cannot necessarily trust the results of either a positive or negative test conclusively. A negative test could simply be a result of the degradation of the antigen resulting in the lack of identification on the test strip, while a positive test can only be confirmed when we know exactly what antigens are binding, which can likely be confirmed with mass spectrometry (however, this is hindered by the manufacturers of the clinical tests who have yet to share the quantities of antibodies they use on the tests — see Loufouma Mbouaka et al. 2020 for further discussion on this). Within our study, the similarities with the genetic test indications are extremely important, as it suggests support for the positive immunoassays, providing some endorsement for the accuracy of the tests, but the application of targeted or shotgun proteomics would contribute significantly to the identification of *Plasmodium* antigens and is an, as yet, unexplored avenue of research for malaria in the past (Cappellini et al. 2018).

### Sample selection implications for biomolecular and genomic analysis

Sample selection on several levels will impact the results of the analyses. First, on the macro-level, we choose a site in Egypt which is known as an endemic area for malaria in antiquity (Malcolm et al. 2007). This was to ensure a higher probability of the presence of *Plasmodium* within the framework of this study, aimed at testing the methods of identification. Secondly, on an individual level, samples were selected for the study based on the presence of *cribra orbitalia*, due to its association with anaemia which has been used as a proxy for the presence of malaria in endemic areas. This was decided as we aimed to primarily test biomolecular methods and used the presence of CO as an indication of a higher likelihood of detecting *Plasmodium* in these individuals; further research will test those with and without macroscopic indications of anaemic conditions. Finally, we have chosen samples from different body parts which contained a significant amount of medullary bone, where blood is produced, believing that these areas would be more likely to conserve either the antigens or the DNA of the *Plasmodium* parasites. Bone samples were used for both molecular approaches, while tooth samples were only used for genomic analysis due to the small quantity of material for analysis. Teeth are generally considered to preserve endogenous pathogen DNA better than most parts of the body (see Spyrou et al. 2019 for a review), which was why they were included in our analysis, as we hoped the preservation would extend to *Plasmodium* DNA; however, this did not seem to be the case. Some of the teeth analysed were still developing (meaning the root apex was not closed in five cases (Table 1) — none showed indications of *plasmodium*, nor good results for endogenous DNA) which could increase risk of contamination from soil; this does not seem to have a bearing on our results (see Table S1). It has been shown in two recent studies (Obaldia et al. 2018; Brito et al. 2020) that the bone marrow represents a major reservoir of a preserved phenotype of *Plasmodium* parasites after death caused by the disease and is an ideal marker for investigating malaria in a clinical setting. This supports our choice of bones with larger blood producing portions for analysis and is reflected in our results, with more bones than teeth providing us with a positive indication for the presence of malaria. In general, we would propose including long bones in future bioarchaeological studies since they seem to provide an effective source for identifying the presence of *Plasmodium* DNA or antigens within archaeological samples. This could prove particularly relevant as frequent requests for destructive analysis of specific parts of the skeleton are making collections curators more cautious about permissions.

### Malaria in Sayala in the Roman and Byzantine periods

The results of our analysis make it difficult to make any sweeping suggestions regarding the presence of malaria in Sayala during the late third to mid sixth centuries AD, as the sample size is rather small and the results were not able to be authenticated. However, we can discuss some of the trends which we have observed here in the context of what is known about the community at Sayala and wider Nubia and southern Egypt. To date, there is no complete site report for the excavations at Sayala; however, there are several preliminary publications which can provide a better understanding of the social hierarchy and community at Sayala in the late Roman–early Byzantine period (i.e. Bietak and Jungwirth 1966; Strouhal and Jungwirth 1971, 1977, 1979, 1980, 1982). There are two types of burial complexes, the more elaborate concentric stone mound style of CI, CII, and CIII, and the simple pit graves of cemetery N which contained a higher proportion of the individuals under 10 years of age (Strouhal and Jungwirth 1971). Strouhal and Jungwirth have suggested that this reflects different treatments for children and some women (as there were fewer females than expected by the authors in the C cemeteries) (Strouhal and Jungwirth 1980). Sayala was a border town of the Roman Empire, with a community of indigenous Nubians, predominantly from the Blemyer (Blemmyes) tribe^1^ (Bietak and Jungwirth 1966: 468; Strouhal and Jungwirth 1979). The excavations did not reveal a settlement area, suggesting that the Roman period habitation was either destroyed by an earlier construction of the Aswan Dam or the building materials from the site were utilised by the modern inhabitants of Sayala; however, ceramics and other material culture indicate a large and varied population (Bietak and Jungwirth 1966: 468). The only non-mortuary structures identified are Roman wine taverns which support the use of Sayala as a frontier town, a stopping point along the gold route from Nubia to the north, with similarities to Wadi-el-Arab (Bietak and Jungwirth 1966: 469). Malaria has been attested in ancient Egyptian mummified remains dating back to c. 1500 BC (Nerlich et al. 2008). While similar findings from Nubia are so far missing, it is assumed that the spread of the disease into Egypt from the more southern regions of Africa would have occurred via the Nile Valley route; thus, its presence in Egypt also presupposes a presence of the disease in the Middle Nile Valley from at least the 2nd millennium BC onwards (Malcolm et al. 2007). More recently, an outbreak in the Aswan area in 2014 provides evidence of the continued hospitable environment for the vector and human host interaction (Kandeel et al. 2016).

Strouhal’s palaeopathological assessment suggests the presence of haemopoietic and/or metabolic diseases within the community (Strouhal unpublished: sections 11.5–11.6). While our re-analysis of the pathological lesions on the ten individuals within this study does not bring to light any definitive pathologies associated with the possible presence of malaria, it has shown that the individuals who suffered from CO consistently also demonstrated other systemic non-specific pathological changes. Smith-Guzmán presents the argument that the presence of CO represents a skeletal response to anaemia (of any aetiology) and that ‘although there are many factors that could have potentially contributed to the overall anaemia seen in the human skeletal remains of ancient Egypt, malarial infection has been shown to have a major synergistic effect with other factors to increase overall anaemia levels; thus, would have arguably raised the overall frequencies of *cribra orbitalia*’ (2015a, p. 1). Therefore, if we consider that Strouhal observed that 68.6% of the infants and children with intact orbits and 14.5% of adults with intact orbits display *cribra orbitalia*, it is possible that malaria was more frequent than our biomolecular analyses have revealed. Certainly, it seems that this population faced nutritional or haemopoietic stresses which is consistent with other Nile Valley populations around this time (Groff and Dupras 2019; Scheidel 2001).

Overall, between four and six of the ten (40–60%) individuals examined presented a positive result of either both or one biomolecular test, and of these, five of seven (71%) of the genetically female skeletons tested were positive, while only one of three (33%) of the male skeletons are. The greater number of females in the sample group reflects a blind aspect of the study, as most of the individuals could not be osteologically assessed for sex, and it was only genetically identified later; this implies that more females than males displayed osteological markers of possible anaemia (supported by Stouhal’s assessment of all the adult individuals, noted above). Clinical research indicates that children and pregnant women are at the greatest risk of acquiring malarial anaemia (Menendez et al. 2000; White 2018), which may have bearing firstly, on our sample selection, and also on the indications of the presence of malaria in the biomolecular analyses. While our sample is too small to make statements regarding malarial infection based on sex at Sayala, it perhaps is useful for future research to note the discrepancy in the expression of anaemia symptoms between the sexes when considering sample selection. Most research exploring the differences in expression of malaria has determined that economic, socio-cultural, and personal (biological) determinants are the primary factors resulting in variations in infection and treatment (Burns and Boyce 2015; Vlassoff and Bonilla 1994: 41). While treatment was not available in Sayala in antiquity, there would have almost certainly been economic and socio-cultural factors (such as gender-divided activities and/or diet, habitation location in relation to standing water, various aspects of diet and possibly life-stage or age-related changes in food or activities) which would have impacted the infection rates amongst the different groups. In an attempt to ensure we covered a spectrum of the community in Sayala we sampled from the different cemeteries surrounding the site; however, while there is a suggested status difference between the C cemeteries and the N cemetery, only one individual from cemetery N was analysed; therefore, it is not possible to make any statements regarding differences amongst social groups from Sayala (Bietak and Jungwirth 1966).

Our four individuals who provide the most persuasive indications for the presence of malaria in the Sayala population (with two positive, though unauthenticated, biomolecular tests) represent both sexes, albeit disproportionately female, and predominately are over the age of 12 years-at-death. Sayala was a large population situated on the banks of the Nile, and there are still questions regarding the nature of the community and the social structure associated with it, which, once answered, may help to understand the prevalence of malaria within this community. Our results, across the three methods of analysis, indicate that there is consistency across biomolecular methods, but further refinement is still needed to be able to authenticate the results for precise identification of malaria in the past. It is only when this is possible that prevalence and distribution of the disease and the different species of malaria can be discussed, alongside the impact such an endemic disease would have had on the community.

## Conclusion

The results of this study, in which four of the ten individuals sampled provided matching positive results for both malarial antigens and indications of *Plasmodium* DNA, suggest that there is great promise in applying multiple analyses to an archaeologically derived skeletal collection. At the moment, the limitations predominantly relate to the thresholds imposed by the specificity and sensitivity of the tests applied; thus, with continual improvements to the technology, both these methods could have a great impact on our understanding of disease prevalence and its effect on populations in the past. The methods of DNA extraction and analysis are improving with great speed, and specific DNA fragments are consistently identified with greater precision from very ancient remains. Still, however, there is a limit to analysing ancient DNA when the material is highly degraded. Likewise, although the use of RDTs in identifying malaria in the past has had a relatively long history, there still remain issues with specificity and identification of the binding antibodies. Thus, while a false positive is not likely in aDNA analyses, it is certainly still possible with the RDTs, and conversely, a negative result in either analysis is not a conclusive negative. Importantly, proteins tend to be more stable than DNA in ancient human remains. Therefore, focus of future analyses should be put on the identification of specific *Plasmodium* proteins to avoid false positive diagnostics. Overall, there was no evidence from the palaeopathological analysis of the skeletal material of distinctive consistent osseous changes between individuals who subsequently showed positive genetic and immunological results for the presence of *Plasmodium* and those who did not.

We suggest, as a result of our investigation, that bones, with good preservation of the medullary bone, are likely an effective sample source in the search for malaria and that collaboration between multiple strands of biomolecular testing contributes to the confirmation and success of the results. Irrefutable evidence of the presence of malaria through macroscopic observations is the goal of palaeopathologists to avoid destructive analyses; however, this will be most effectively realised through rigorous comparison amongst methods on a skeletal collection which is well-preserved with documented malaria cases. As we have noted above, in terms of biomolecular analyses, the survivability of proteins encourages further exploration into immunological assays in particular. It is only when the results are consistent and replicable that malaria will be identified with certainty in ancient remains, and the evolutionary history of this significant disease can be explored thoroughly as researchers seek to create a vaccine. Our research provides new insight and shows the complexity of investigating diseases in the past through multidisciplinary approaches; it also demonstrates the benefit of combining different techniques in the refinement of the process of identification of malaria in ancient human remains.

## A.Supplementary information

ESM 1 (DOCX 14 kb)

ESM 2 (DOCX 17 kb)

ESM 3 (XLSX 30 kb)

ESM 4 (JPG 5321 kb)

ESM 5 (JPG 4632 kb)

ESM 6 (JPG 5698 kb)

ESM 7 (JPG 5139 kb)

ESM 8 (JPG 1334 kb)

ESM 9 (JPG 1363 kb)

ESM 10 (JPG 1349 kb)

ESM 11 (JPG 1028 kb)

ESM 12 (JPG 1341 kb)

ESM 13 (JPG 4192 kb)

ESM 14 (JPG 174 kb)

ESM 15 (DOCX 270 kb)

ESM 16 (DOCX 120 kb)

ESM 17 (DOCX 823 kb)

ESM 18 (DOCX 166 kb)

## Data Availability

aDNA data are available from the European Nucleotide Archive under accession no. PRJEB43969. The samples from Sayala were selected based on an unpublished report by Eugen Strouhal which is currently being edited for publication by the Austrian Academy of Sciences, through a grant from the British Museum.

## Footnotes

1 It must be noted here that all the assignation of ethnicity and race was based on antiquated methods no longer accepted in anthropological analysis. The theories behind these methods have racist origins and while we do not believe that the authors intended any racist motives in this case, and were simply applying what was an accepted scientific method of the 1960s, we would be remiss to mention their results without decrying the racist roots of these methods. For further discussion on the assignation of race in anthropology, see, for example, Reynolds and Lieberman (1996), Sauer et al. (2016), and Stojanowski (2019).

## References

[CR1] Agarwal SC (2016). Bone morphologies and histories: life course approaches in bioarchaeology. Am J Phys Anthropol.

[CR2] Alano P (2017). The emerging role of the human bone marrow as a privileged developmental niche for the transmission stages of the malaria parasite Plasmodium falciparum. Commentary Ann Ist Super Sanita.

[CR3] Ali H, Ahsan T, Mahmood T, Feroze Bakht S, Umer Farooq M, Ahmed N (2008). Parasite density and the spectrum of clinical illness in falciparum malaria. J Coll Phys Surg Pakistan.

[CR4] Al-Khafif GD, El-Banna R, Khattab N, Rashed TG, Dahesh S (2018). The immunodetection of non-falciparum malaria in ancient Egyptian bones (Giza Necropolis). Biomed Res Int.

[CR5] Altschul SF, Gish W, Miller W, Myers EW, Lipman DJ (1990). Basic local alignment search tool. J Mol Biol.

[CR6] Andrews RM, Kubacka I, Chinnery PF, Lightowlers RN, Turnbull DM, Howell N (1999). Reanalysis and revision of the Cambridge reference sequence for human mitochondrial DNA. Nat Genet.

[CR7] Angel JL (1966). Porotic hyperostosis, anemias, malarias, and marshes in the prehistoric Eastern Mediterranean. Science.

[CR8] Angel JL (1978). Porotic hyperostosis in the eastern Mediterranean. MCV/Q, Medic Coll Virginia Quarter.

[CR9] Arrow KJ, Panosian CB, Gelbrand H (eds) (2004) Saving lives, buying time: economics of malaria drugs in an age of resistance. The National Academies Press, Washington, D.C. http://www.nap.edu/catalog/11017.html. 25009879

[CR10] Aufderheide AC, Rodríguez-Martín C (1998) The Cambridge encyclopaedia of human paleopathology. Cambridge University Press

[CR11] Bartoloni A, Strohmeyer M, Sabatinelli G, Benucci M, Serni U, Paradisi F (1998). False Positive *Para*Sight™-F test for malaria patients with rheumatoid factor. Trans R Soc Trop Med Hyg.

[CR12] Berens-Riha N, Sinicina I, Fleischmann E, Löscher T (2009). Comparison of different methods for delayed post-mortem diagnosis of falciparum malaria. Malar J.

[CR13] Bianucci R, Mattutino G, Lallo R, Charlier P, Jouin-Spriet H, Peluso A, Higham T, Torre C, Massa ER (2008). Immunological evidence of Plasmodium falciparum infection in an Egyptian child mummy from the Early Dynastic Period. J Archaeol Sci.

[CR14] Bianucci R, Araujo A, Pusch CM, Nerlich AG (2015). The identification of malaria in paleopathology – an in-depth assessment of the strategies to detect malaria in ancient remains. Acta Trop.

[CR15] Bietak M, Jungwirth J (1966). Die Österreichischen Grabungen in Ägyptisch-Nubien im Herbst 1965. Ann Naturhist Mus Wien.

[CR16] Bollongino R, Tresset A, Vigne J-D (2008). Environment and excavation: pre-lab impacts on ancient DNA analyses. C.R. Palevol.

[CR17] Bolton-Maggs P, Thomas A (2008) Disorders of the blood and bone marrow. In: Forfar and Arneil’s textbook of pediatrics, vol 6, p 1059

[CR18] Brickley MB (2018). Cribra orbitalia and porotic hyperostosis: a biological approach to diagnosis. Am J Phys Anthropol.

[CR19] Brickley M, McKinley JI (2004) Guidelines to the standards for recording human remains, IFA paper, vol 7, p 62

[CR20] Brito M, Baro B, Raiol TC, Ayllon-Hermida A, Safe IP, Deroost K, Figueiredo E, Costa AG, Armengol M, Sumoy L, Almeida A, Hounkpe BW, De Paula EV, Fernandez-Becerra C, Monteiro WM, Del Portillo HA, Lacerda M (2020) Morphological and transcriptional changes in human bone marrow during natural Plasmodium vivax malaria infections. J Infect Dis 177. 10.1093/infdis/jiaa177 10.1093/infdis/jiaa177 PMC8974851 32556188

[CR21] Brown T, Brown K (2011) Biomolecular archaeology: an introduction. Wiley-Blackwell

[CR22] Bruce-Chwatt LJ (1988) History of malaria from prehistory to eradication. In: Wernsdorfer WH, McGregor I (eds) Malaria: principles and practice of malariology. Churchill Livingstone, Edinburgh, pp 1–59

[CR23] Buchfink B, Xie C, Huson DH (2015). Fast and sensitive protein alignment using DIAMOND. Nat Methods.

[CR24] Buikstra JE (ed) (2019) Ortner’s identification of pathological conditions in human skeletal remains. Academic Press

[CR25] Buikstra JE, Ubelaker DH (1994) Standards for data collection from human remains. Arkansas Archaeological Survey, Fayetteville

[CR26] Burns, K. & Boyce, C. (2015). Gender and malaria: making the investment case for programming that addresses the specific vulnerabilities and needs of both males and females who are affected by or at risk of malaria. UNDP Discussion Paper, December 2015. https://www.undp.org/content/dam/undp/library/HIV-AIDS/Gender%20HIV%20and%20Health/Discussion%20Paper%20Gender_Malaria.pdf.

[CR27] Calarco L, Barratt J, Ellis J (2020). Detecting sequence variants in clinically important protozoan parasites. Int J Parasitol.

[CR28] Caminade C, Kovats S, Rocklov J, Tompkins AM, Morse AP, Colón-González FJ, Stenlund H, Martens P, Lloyd SJ (2014). Impact of climate change on global malaria distribution. Proc Natl Acad Sci U S A.

[CR29] Cappellini E, Prohaska A, Racimo F, Welker F, Pedersen MW, Allentoft ME, de Barros Damgaard P, Gutenbrunner P, Dunne J, Hammann S, Roffet-Salque M, Ilardo M, Moreno-Mayar JV, Wang Y, Sikora M, Vinner L, Cox J, Evershed RP, Willerslev E (2018). Ancient biomolecules and evolutionary inference. Annu Rev Biochem.

[CR30] Carter R, Mendis KN (2002). Evolutionary and historical aspects of the burden of malaria. Clin Microbiol Rev.

[CR31] Cole G, Waldron T (2019). Cribra orbitalia: dissecting an ill-defined phenomenon. Int J Osteoarchaeol.

[CR32] Cox MD (2018) Malaria in the prehistoric Caribbean: the hunt for hemozoin, Electronic Theses and Dissertations. Paper 2926. 10.18297/etd/2926

[CR33] Dabney J, Meyer M, Pääbo S (2013). Ancient DNA damage. Cold Spring Harb Perspect Biol.

[CR34] Dalrymple U, Arambepola R, Gething PW, Cameron E (2018). How long do rapid diagnostic tests remain positive after anti-malarial treatment?. Malar J.

[CR35] Damgaard PB, Margaryan A, Schroeder H, Orlando L, Willerslev E, Allentoft ME (2015). Improving access to endogenous DNA in ancient bones and teeth. Sci Rep.

[CR36] de Dios T, van Dorp L, Gelabert P, Carøe C, Sandoval-Velasco M, Fregel R, Escosa R, Aranda C, Huijben S, Balloux F, Gilbert M, Lalueza-Fox C (2019). Genetic affinities of an eradicated European Plasmodium falciparum strain. Microbial Genom.

[CR37] De Sanctis V, Kattamis C, Canatan D, Soliman AT, Elsedfy H, Karimi M, Daar S, Wali Y, Yassin M, Soliman N, Sobti P, Al Jaouni S, El Kholy M, Fiscina B, Angastiniotis M (2017). β-thalassemia distribution in the old world: an ancient disease seen from a historical standpoint. Mediterr J Hematol Infect Diseases.

[CR38] Demarchi B, Hall S, Roncal-Herrero T, Freeman CL, Woolley J, Crisp MK, Wilson J, Fotakis A, Fischer R, Kessler BM, Rakownikow Jersie-Christensen R, Olsen JV, Haile J, Thomas J, Marean CW, Parkington J, Presslee S, Lee-Thorp J, Ditchfield P, Hamilton JF (2016). Protein sequences bound to mineral surfaces persist into deep time. eLife.

[CR39] DeWitte SN, Stojanowski CM (2015). The osteological paradox 20 years later: past perspectives, future directions. J Archaeol Res.

[CR40] Donoghue HD, Spigelman M, Zias J, Gernaey-Child AM, Minnikin DE (1998). Mycobacterium tuberculosis complex DNA in calcified pleura from remains 1400 years old. Lett Appl Microbiol.

[CR41] Dutour O (2016). Paleopathology of human infections: old bones, antique books, ancient and modern molecules. Microbiol Spectrum.

[CR42] Farfour E, Charlotte F, Settegrana C, Miyara M, Buffet P (2012). The extravascular compartment of the bone marrow: a niche for Plasmodium falciparum gametocyte maturation?. Malar J.

[CR43] Fornaciari G, Giuffra V, Ferroglio E, Gino S, Bianucci R (2010). Plasmodium falciparum immunodetection in bone remains of members of the Renaissance Medici family (Florence, Italy, sixteenth century). Trans R Soc Trop Med Hyg.

[CR44] Galatas B, Bassat Q, Mayor A (2016). Malaria parasites in the asymptomatic: looking for the hay in the haystack. Trends Parasitol.

[CR45] Galaway F, Yu R, Constantinou A, Prugnolle F, Wright GJ (2019) Resurrection of the ancestral RH5 invasion ligand provides a molecular explanation for the origin of P. falciparum malaria in humans. PLoS Biology, 17(10): e3000490. 10.1371/journal.pbio.3000490 PMC6793842 31613878

[CR46] Garcia LS (2010). Malaria. Clin Lab Med.

[CR47] Gelabert P, Sandoval-Velasco M, Olalde I, Fregel R, Rieux A, Escosa R, Aranda C, Paaijmans K, Mueller I, Gilbert MT, Lalueza-Fox C (2016). Mitochondrial DNA from the eradicated European Plasmodium vivax and P. falciparum from 70-year-old slides from the Ebro Delta in Spain. Proc Natl Acad Sci U S A.

[CR48] Gowland R, Garnsey P (2010) Skeletal evidence for health, nutritional status and malaria in Rome and the empire. Archaeological Approaches to Mobility and Diversity in the Roman Empire, Roman Diasporas, pp 131–156

[CR49] Gowland RL, Western AG (2012). Morbidity in the marshes: using spatial epidemiology to investigate skeletal evidence for Malaria in Anglo-Saxon England (AD 410-1050). Am J Phys Anthropol.

[CR50] Grobusch MP, Alpermann U, Schwenke S, Jelinek T, Warhurst DC (1999). False-positive rapid tests for malaria in patients with rheumatoid factor. Lancet.

[CR51] Groff AT, Dupras TL (2019) Isotopes, migration and sex: investigating the mobility of the frontier inhabitants of Roman Egypt. In: Tica CI, Martin DL (eds) Bioarchaeology of frontiers and borderlands. University Press of Florida, Gainesville, pp 83–106

[CR52] Gunawardena S, Karunaweera ND (2015). Advances in genetics and genomics: use and limitations in achieving malaria elimination goals. Pathog Global Health.

[CR53] Hartl DL (2004) The origin of malaria: mixed messages from genetic diversity. Nature Reviews Microbiology, 2(1):15–22. 10.1038/nrmicro795 15035005

[CR54] Haldane JBS (1949). Disease and evolution. La Ricerca Scient.

[CR55] Hawass Z, Gad YZ, Ismail S, Khairat R, Fathalla D, Hasan N, Ahmed A, Elleithy H, Ball M, Gaballah F, Wasef S, Fateen M, Amer H, Gostner P, Selim A, Zink A, Pusch CM (2010). Ancestry and pathology in King Tutankhamun’s family. JAMA.

[CR56] Hay SI, Guerra CA, Tatem AJ, Noor AM, Snow RW (2004). The global distribution and population at risk of malaria: past, present, and future. Lancet Infect Dis.

[CR57] Hedrick PW (2012). Resistance to malaria in humans: the impact of strong, recent selection. Malar J.

[CR58] Hempelmann E, Krafts K (2013). Bad air, amulets and mosquitoes: 2,000 years of changing perspectives on malaria. Malar J.

[CR59] Hens SM, Godde K, Macak KM (2019). Iron deficiency anemia, population health and frailty in a modern Portuguese skeletal sample. PLoS One.

[CR60] Hofreiter M, Paijmans JL, Goodchild H, Speller CF, Barlow A, Fortes GG, Thomas JA, Ludwig A, Collins MJ (2015). The future of ancient DNA: technical advances and conceptual shifts. Bioessays.

[CR61] Huson DH, Beier S, Flade I, Górska A, El-Hadidi M, Mitra S, Ruscheweyh HJ, Tappu R (2016). MEGAN community edition - interactive exploration and analysis of large-scale microbiome sequencing data. PLoS Comput Biol.

[CR62] James W, Johnson RJ, Speakman JR, Wallace DC, Frühbeck G, Iversen PO, Stover PJ (2019). Nutrition and its role in human evolution. J Intern Med.

[CR63] Jónsson H, Ginolhac A, Schubert M, Johnson PL, Orlando L (2013). mapDamage2. 0: fast approximate Bayesian estimates of ancient DNA damage parameters. Bioinformatics.

[CR64] Kandeel A, Haggag AA, Abo El Fetouh M, Naiel M, Refaey SA (2016). Control of malaria outbreak due to Plasmodium vivax in Aswan governorate, Egypt. EMHJ-East Mediterran Health J.

[CR65] Kendall C, Eriksen AMH, Kontopoulos I, Collins MJ, Turner-Walker G (2018). Diagenesis of archaeological bone and tooth. Palaeogeogr Palaeoclimatol Palaeoecol.

[CR66] Kwiatkowski DP (2005). How malaria has affected the human genome and what human genetics can teach us about malaria. Am J Hum Genet.

[CR67] Lafferl H, Kandel K, Pichler H (1997). False positive dipstick test for malaria. N Engl J Med.

[CR68] Lalremruata A, Ball M, Bianucci R, Welte B, Nerlich AG, Kun JF, Pusch CM (2013). Molecular identification of falciparum malaria and human tuberculosis co-infections in mummies from the Fayum depression (Lower Egypt). PLoS One.

[CR69] Langmead B, Salzberg SL (2012). Fast gapped-read alignment with Bowtie 2. Nat Methods.

[CR70] Larsen CS (2018). Bioarchaeology in perspective: from classifications of the dead to conditions of the living. Am J Phys Anthropol.

[CR71] Lee M, Maruyama K, Fujita Y, Konishi A, Lelliott PM, Itagaki S, Horii T, Lin JW, Khan SM, Kuroda E, Akira S, Ishii KJ, Coban C (2017). Plasmodium products persist in the bone marrow and promote chronic bone loss. Sci Immunol.

[CR72] Lewis M (2018) Paleopathology of children: identification of pathological conditions in the human skeletal remains of non-adults. Elsevier Academic Press

[CR73] Li H, Durbin R (2010) Fast and accurate long-read alignment with Burrows–Wheeler transform. Bioinformatics, 26(5):589–595. 10.1093/bioinformatics/btp698 PMC2828108 20080505

[CR74] Li H, Handsaker B, Wysoker A, Fennell T, Ruan J, Homer N, Marth G, Abecasis G, Durbin R, 1000 Genome Project Data Processing Subgroup (2009). The sequence alignment/map format and SAMtools. Bioinformatics (Oxford, England).

[CR75] Loufouma Mbouaka A, Binder M, Noedl H, Gamble M (2020). Evaluation of rapid diagnostic tests and enzyme linked immunoassay in the detection of malaria in ancient human remains. J Archaeol Sci.

[CR76] Loy DE, Liu W, Li Y, Learn GH, Plenderleith LJ, Sundararaman SA, Sharp PM & Hahn, BH (2017) Out of Africa: origins and evolution of the human malaria parasites Plasmodium falciparum and Plasmodium vivax. Int J Parasit 47(2–3):87–97. 10.1016/j.ijpara.2016.05.008 10.1016/j.ijpara.2016.05.008 PMC5205579 27381764

[CR77] Lu J, Salzberg SL (2018). Removing contaminants from databases of draft genomes. PLoS Comput Biol.

[CR78] Malcolm CA, Welsby DA, El Sayed BB (2007) SIT for the malaria vector Anopheles arabiensis in Northern State, Sudan: an historical review of the field site. In: Vreysen MJB, Robinson AS, Hendrichs J (eds) Area-Wide Control of Insect Pests. Springer, Dordrecht. 10.1007/978-1-4020-6059-5_34

[CR79] Marciniak S (2016) Chapter 6 - hunting for pathogens: ancient DNA and the historical record. In: Beyond the Bones. Academic Press, pp 81–100. 10.1016/B978-0-12-804601-2.00006-5

[CR80] Marciniak S, Prowse TL, Herring DA, Klunk J, Kuch M, Duggan AT, Bondioli L, Holmes EC, Poinar HN (2016). Plasmodium falciparum malaria in 1st-2nd century CE southern Italy. Curr Biol.

[CR81] Marciniak S, Herring DA, Sperduti A, Poinar HN, Prowse TL (2018). A multi-faceted anthropological and genomic approach to framing Plasmodium falciparum malaria in Imperial period central-southern Italy (1st–4th c. CE). J Anthropol Archaeol.

[CR82] Menendez C, Fleming AF, Alonso PL (2000). Malaria-related anaemia. Parasitol Today.

[CR83] Meyer M, Kircher M (2010). Illumina sequencing library preparation for highly multiplexed target capture and sequencing. Cold Spring Harb Protoc.

[CR84] Miller RL, Ikram S, Armelagoss GJ, Walker R, Harers B, Shiffi CJ, Baggett D, Carrigan M, Maret SM (1994). Diagnosis of Plasmodium falciparum infections in mummies using the rapid manual ParaSightTM-F test. Trans R Soc Trop Med Hyg.

[CR85] Mishra B, Samantaray JC, Kumar A, Mirdha BR (1999). Study of false positivity of two rapid antigen detection tests for diagnosis of Plasmodium falciparum malaria [3]. J Clin Microbiol.

[CR86] Mitchell, P.D. & Brickley, M. (eds). (2017). Updated guidelines to the standards for recording human remains. Chartered Institute for Archaeologists/British Association for Biological Anthropology and Osteoarchaeology: Reading 2017. Available from: http://www.babao.org.uk/assets/Uploads-to-Web/14-Updated-Guidelines-to-the-Standards-for-Recording-Human-Remains-digital.pdf.

[CR87] Moody AH, Chiodini PL (2002). Non-microscopic method for malaria diagnosis using OptiMAL IT, a second-generation dipstick for malaria pLDH antigen detection. Br J Biomed Sci.

[CR88] Murray CJ, Rosenfeld LC, Lim SS, Andrews KG, Foreman KJ, Haring D, Fullman N, Naghavi M, Lozano R, Lopez AD (2012). Global malaria mortality between 1980 and 2010: a systematic analysis. Lancet (London, England).

[CR89] NCBI Resource Coordinators (2017). Database resources of the National Center for Biotechnology Information. Nucleic Acids Res.

[CR90] Nerlich A (2016). Paleopathology and paleomicrobiology of malaria. Microbiol Spectrum.

[CR91] Nerlich AG, Schraut B, Dittrich S, Jelinek T, Zink AR (2008). Plasmodium falciparum in ancient Egypt. Emerg Infect Dis.

[CR92] O’Donnell L, Hill EC, Anderson ASA, Edgar HJ (2020). Cribra orbitalia and porotic hyperostosis are associated with respiratory infections in a contemporary mortality sample from New Mexico. Am J Phys Anthropol.

[CR93] Oaks SC Jr, Mitchell VS, Pearson GW, Carpenter C (eds) (1991) Malaria: obstacles and opportunities, Institute of Medicine (US) Committee for the Study on Malaria Prevention and Control. National Academies Press (US) Available from: https://www.ncbi.nlm.nih.gov/books/NBK234333/25121285

[CR94] Obaldia N, Meibalan E, Sa JM, Ma S, Clark MA, Mejia P, Moraes Barros RR, Otero W, Ferreira MU, Mitchell JR, Milner DA, Huttenhower C, Wirth DF, Duraisingh MT, Wellems TE, Marti M (2018). Bone barrow is a major parasite reservoir in Plasmodium vivax infection. mBio.

[CR95] Ondov BD, Bergman NH, Phillippy AM (2011). Interactive metagenomic visualization in a web browser. BMC Bioinformat.

[CR96] Ortner DJ (2003) Identification of pathological conditions in human skeletal remains, 2nd edn. London Academic Press

[CR97] Oxenham MF, Cavill I (2010). Porotic hyperostosis and Cribra orbitalia: the erythropoietic response to iron-deficiency anaemia. Anthropol Sci.

[CR98] Pääbo S (1985). Molecular cloning of ancient Egyptian mummy DNA. Nature.

[CR99] Pääbo S (1989). Ancient DNA: extraction, characterization, molecular cloning, and enzymatic amplification. Proc Natl Acad Sci.

[CR100] Pääbo S, Higuchi RG, Wilson AC (1989). Ancient DNA and the polymerase chain reaction: the emerging field of molecular archaeology (Minireview). J Biol Chem.

[CR101] Pääbo S, Poinar H, Serre D, Jaenicke-Després V, Hebler J, Rohland N, Kuch M, Krause K, Vigilant L, Hofreiter M (2004). Genetic analyses from ancient DNA. Annu Rev Genet.

[CR102] Piperaki ET, Daikos GL (2016). Malaria in Europe: emerging threat or minor nuisance?. Clin Microbiol Infect.

[CR103] Pruvost M, Schwarz R, Correia VB, Champlot S, Braguier S, Morel N, Fernandez-Jalvo Y, Grange T, Geigl EM (2007). Freshly excavated fossil bones are best for amplification of ancient DNA. Proc Natl Acad Sci U S A.

[CR104] Rabino Massa E, Cerutti N, Savoia MD (2000). Malaria in ancient Egypt: paleoimmunological investigation on predynastic mummified remains. Chungará (Arica).

[CR105] Renaud G, Slon V, Duggan AT, Kelso J (2015). Schmutzi: estimation of contamination and endogenous mitochondrial consensus calling for ancient DNA. Genome Biol.

[CR106] Reynolds LT, Lieberman L (1996) Race and other misadventures: essays in honor of Ashley Montagu in his ninetieth year. General Hall Inc.

[CR107] Rivera F, Mirazón Lahr M (2017). New evidence suggesting a dissociated etiology for Cribra orbitalia and porotic hyperostosis. Am J Phys Anthropol.

[CR108] Rohland N, Siedel H, Hofreiter M (2010). A rapid column-based ancient DNA extraction method for increased sample throughput. Mol Ecol Resour.

[CR109] Rohland N, Glocke I, Aximu-Petri A, Meyer M (2018). Extraction of highly degraded DNA from ancient bones, teeth and sediments for high-throughput sequencing. Nat Protoc.

[CR110] Rook GA, Raison CL, Lowry CA (2014). Microbial ‘old friends’, immunoregulation and socioeconomic status. Clin Exp Immunol.

[CR111] Rosenbloom KR, Armstrong J, Barber GP, Casper J, Clawson H, Diekhans M, Dreszer TR, Fujita PA, Guruvadoo L, Haeussler M, Harte RA, Heitner S, Hickey G, Hinrichs AS, Hubley R, Karolchik D, Learned K, Lee BT, Li CH, Miga KH, Nguyen N, Paten B, Raney BJ, Smit AFA, Speir ML, Zweig AS, Haussler D, Kuhn RM, Kent WJ (2015). The UCSC Genome Browser database: 2015 update. Nucleic Acids Res.

[CR112] Sallares R (2002) Malaria and Rome: a history of malaria in Ancient Italy. Oxford University Press. 10.1093/acprof:oso/9780199248506.001.0001

[CR113] Sallares R, Gomzi S (2001). Biomolecular archaeology of malaria. Anc Biomol.

[CR114] Sauer NJ, Wankmiller JC, Hefner JT (2016) The assessment of ancestry and the concept of race. In: Blau S, Ubelaker DH (eds) Handbook of forensic anthropology and archaeology, 2nd edn. Routledge, New York and London, pp 243–260

[CR115] Schaefer M, Black S, Scheuer L (2009) Juvenile osteology. Academic Press, San Diego. 10.1016/B978-0-12-374635-1.X0001-X

[CR116] Scheidel, W. (2001). Death on the Nile: disease and the demography of Roman Egypt. Mnemosyne, Supplements, Volume: 228 and Mnemosyne, Supplements, History and Archaeology of Classical Antiquity, Volume: 228. Brill. 10.1163/9789004350946

[CR117] Scherf A, Pouvelle B, Buffet PA, Gysin J (2001). Molecular mechanisms of Plasmodium falciparum placental adhesion. Cell Microbiol.

[CR118] Schuenemann VJ, Peltzer A, Welte B, van Pelt WP, Molak M, Wang CC, Furtwängler A, Urban C, Reiter E, Nieselt K, Teßmann B, Francken M, Harvati K, Haak W, Schiffels S, Krause J (2017). Ancient Egyptian mummy genomes suggest an increase of sub-Saharan African ancestry in post-Roman periods. Nat Commun.

[CR119] Setzer TJ (2014). Malaria detection in the field of paleopathology: a meta-analysis of the state of the art. Acta Trop.

[CR120] Skoglund P, Storå J, Götherström A, Jakobsson M (2013). Accurate sex identification of ancient human remains using DNA shotgun sequencing. J Archaeol Sci.

[CR121] Smalley ME, Abdalla S, Brown J (1981). The distribution of Plasmodium falciparum in the peripheral blood and bone marrow of Gambian children. Trans R Soc Trop Med Hyg.

[CR122] Smith-Guzmán NE (2015). Cribra orbitalia in the ancient Nile Valley and its connection to malaria. Int J Paleopathol.

[CR123] Smith-Guzmán NE (2015). The skeletal manifestation of malaria: An epidemiological approach using documented skeletal collections. Am J Phys Anthropol.

[CR124] Soren D, Fenton T, Birkby W (1995). The late Roman infant cemetery near Lugnano in Teverina, Italy: some implications. J Paleopathol.

[CR125] Spyrou MA, Bos KI, Herbig A, Krause J (2019). Ancient pathogen genomics as an emerging tool for infectious disease research. Nat Rev Genet.

[CR126] Stojanowski CM (2019) Ancient migrations: biodistance, genetics and the persistence of typological thinking. In: Buikstra JE (ed) Bioarchaeologists speak out: deep time perspectives on contemporary issues, Bioarchaeology and Social Theory Series. Springer, Switzerland, pp 181–200. 10.1007/978-3-319-93012-1_8

[CR127] Strouhal E (1992). Anthropology of the Christian population at Sayala (Egyptian Nubia, 6th-11th century A.D.): preliminary report. Anthropologie (1962-).

[CR128] Strouhal, E. (unpublished). Anthropological examination of Late Roman–Early Byzantine cemeteries from Sayala, Egyptian Nubia. Reports of the Austrian National Committee of the UNESCO Action for Saveguarding Nubian Monuments Publication. Austrian Academy of Sciences Publishing House.

[CR129] Strouhal E, Jungwirth J (1971). Anthropological problems of the Middle Empire and late Roman Sayala. Mitteilungen der Anthropologischen Gesellschaft (Wien).

[CR130] Strouhal E, Jungwirth J (1977). Ein verkalktes Myoma uteri aus der späten Römerzeit in Ägyptisch-Nubien. Mitteilungen der Anthropologischen Gesellschaft (Wien).

[CR131] Strouhal E, Jungwirth J (1979). Paleogenetics of the late Roman-early Byzantine cemeteries at Sayala, Egyptian Nubia. J Hum Evol.

[CR132] Strouhal E, Jungwirth J (1980). Paleopathology of the late Roman-early Byzantine cemeteries at Sayala, Egyptian Nubia. J Hum Evol.

[CR133] Strouhal E, Jungwirth J (1982) Traumatism in the Late Roman-Early Byzantine cemeteries at Sayala, Egyptian Nubia. In: 22nd Anthropological Congress of Ales Hrdlicka-Universitas Carolina Pragensis, pp 459–461

[CR134] Stuart-Macadam P (1987). Porotic hyperostosis: new evidence to support the anemia theory. Am J Phys Anthropol.

[CR135] Stuart-Macadam PL (1991) Anemia in Roman Britain: Poundbury camp. In: Bush H, Zvelebil M (eds) Health in past societies: biocultural interpretations of human skeletal remains in archaeological contexts. British Archaeological Research International Series, Oxford, pp 101–113

[CR136] Stuart-Macadam P (1992). Porotic hyperostosis: a new perspective. Am J Phys Anthropol.

[CR137] Tangpukdee N, Krudsood S, Kano S, Wilairatana P (2012). Falciparum malaria parasitemia index for predicting severe malaria. Int J Lab Hematol.

[CR138] Tayles N (1996). Anemia, genetic diseases, and malaria in prehistoric mainland Southeast Asia. Am J Phys Anthropol.

[CR139] Turner BL, Klaus HD (2016) Biocultural perspectives in bioarchaeology. In: Zuckerman MK, Martin DL (eds) New directions in biocultural anthropology. John Wiley & Sons, Inc., New York, pp 427–451. 10.1002/9781118962954

[CR140] van der Valk T, Pečnerová P, Díez-del-Molino D, Bergström A, Oppenheimer J, Hartmann S, Xenikoudakis G, Thomas JA, Dehasque M, Sağlıcan E, Rabia Fidan F, Barnes I, Liu S, Somel M, Heintzman PD, Nikolskiy P, Shapiro B, Skoglund P, Hofreiter M, Lister AM, Götherström A, Dalén L (2021). Million-year-old DNA sheds light on the genomic history of mammoths. Nature.

[CR141] van Dorp L, Gelabert P, Rieux A, de Manuel M, de Dios T, Gopalakrishnan S, Carøe C, Sandoval-Velasco M, Fregel R, Olalde I, Escosa R, Aranda C, Huijben S, Mueller I, Marquès-Bonet T, Balloux F, Gilbert M, Lalueza-Fox C (2020). Plasmodium vivax malaria viewed through the lens of an eradicated European strain. Mol Biol Evol.

[CR142] Vlassoff C, Bonilla E (1994). Gender-related differences in the impact of tropical diseases on women: what do we know?. J Biosoc Sci.

[CR143] Wadsworth C, Procopio N, Anderung C, Carretero JM, Iriarte E, Valdiosera C, Elburg R, Penkman K, Buckley M (2017). Comparing ancient DNA survival and proteome content in 69 archaeological cattle tooth and bone samples from multiple European sites. J Proteome.

[CR144] Walker PL, Bathurst RR, Richman R, Gjerdrum T, Andrushko VA (2009). The causes of porotic hyperostosis and cribra orbitalia: a reappraisal of the iron-deficiency-anemia hypothesis. Am J Phys Anthropol.

[CR145] Warinner C, Herbig A, Mann A, Fellows Yates JA, Weiß CL, Burbano HA, Orlando L, Krause J (2017). A robust framework for microbial archaeology. Annu Rev Genomics Hum Genet.

[CR146] Weissensteiner H, Pacher D, Kloss-Brandstätter A, Forer L, Specht G, Bandelt HJ, Kronenberg F, Salas A, Schönherr S (2016). HaploGrep 2: mitochondrial haplogroup classification in the era of high-throughput sequencing. Nucleic Acids Res.

[CR147] White NJ (2018). Anaemia and malaria. Malar J.

[CR148] White T, Folkens P (2005) The human bone manual. Elsevier

[CR149] World Health Organisation (WHO). (2019). World malaria report 2019. Available from: https://www.who.int/publications/i/item/9789241565721. .

[CR150] Wiechmann I, Brandt E, Grupe G (1999). State of preservation of polymorphic plasma proteins recovered from ancient human bones. Int J Osteoarchaeol.

[CR151] Wills B, Ward C, Sáiz-Gómez V, Korenberg C, Phippard J (2014) Conservation of human remains from archaeological contexts. In: Fletcher A, Antoine D, Hill JD (eds) Regarding the dead: human remains in the British Museum. The British Museum, pp 49–74

[CR152] Wongsrichanalai C, Barcus MJ, Muth S, Sutamihardja A, Wernsdorfer WH (2007). A review of malaria diagnostic tools: microscopy and rapid diagnostic test (RDT). Am J Tropic Med Hygiene.

[CR153] Wood JW, Milner GR, Harpending HC, Weiss KM, Cohen MN, Eisenberg LE (1992). The osteological paradox: problems of inferring prehistoric health from skeletal samples [and comments and reply]. Curr Anthropol.

[CR154] World Health Organization (2015) Control and elimination of Plasmodium vivax malaria: a technical brief. World Health Organization

[CR155] Wright LE, Yoder CJ (2003). Recent progress in bioarchaeology: approaches to the osteological paradox. J Archaeol Res.

[CR156] Yakovchuk P, Protozanova E, Frank-Kamenetskii MD (2006). Base-stacking and base-pairing contributions into thermal stability of the DNA double helix. Nucleic Acids Res.

[CR157] Zuckerman M, Turner B, Armelagos G (2012) Evolutionary thought and the rise of the biocultural approach in paleopathology. In: Grauer A (ed) A companion to paleopathology. Wiley-Blackwell, pp 34–58. 10.1002/9781444345940

