# Supplementary material for: The elusive parasite: comparing macroscopic, immunological, and genomic approaches to identifying malaria in human skeletal remains from Sayala, Egypt (third to sixth centuries AD)
Source: Archaeol Anthropol Sci. 2021 Jun 14;13(7):115. doi: 10.1007/s12520-021-01350-z (PMC8202054; doi:10.1007/s12520-021-01350-z)
Supplement: Supplementary file 1 — (DOCX 14 kb) [file 12520_2021_1350_MOESM1_ESM.docx]

Supplementary Online Materials

Brief summary of Loufouma Mbouaka, A., Binder, M., Noedl, H., Gamble, M. (2020). Evaluation of rapid diagnostic tests and Enzyme Linked Immunoassay in the detection of malaria in ancient human remains. Journal of Archaeological Science. 2020: 16: 105118. <https://doi.org/10.1016/j.jas.2020.105118>.

In this study, we evaluated different immunoassay methods, comparing three commercial Rapid Diagnostic Tests (RDTs) alongside Enzyme-linked immunosorbent assay (ELISA) to identify evidence of malaria in archaeologically-derived human skeletal material from an endemic malaria area. The skeletal matter derives from Sayala, Egypt. The three brands of RDT used are: 1) BinaxNow® (Alere Scarborough, Inc, USA) which detects the P. falciparum antigen HRP2, and a pan-malarial antigen, PMA, common to: P. falciparum, P. vivax, P. malariae, and P. ovale; 2) Malaria card test Pv/Pf, DiaSys (DiaSys Diagnostic System GmbH, Holzheim, Germany) which detects the HRP2 of P. falciparum and pLDH specific to P. vivax; and 3) Malaria card test Pan/Pf, DiaSys (DiaSys Diagnostic System GmbH, Holzheim, Germany) for the detection of the HRP2 antigen of P. falciparum, and pLDH for a pan-malarial antigen. ELISA is a plate-based assay technique which is used for detecting and quantifying peptides, proteins, antibodies, and hormones (Engvall and Perlmann 1972). The QDx Malaria Card Tests detected *Plasmodium* antigens in the Sayala samples while BinaxNow® did not; nor did the ELISA. We discuss in detail the possible reasons for this and determined that the two most likely reasons for the different results came down to sensitivity and specificity.

According to a modified version of the published protocol by Bianucci et al., 2008, samples were first cleaned with hydrogen peroxide at 0.3% and then exposed to UV light for disinfection and the samples were crushed with a mortar and pestle. The pulverised bone samples were suspended and mixed in 200 µl of sterile physiological saline solution (Fresenius Physiological Saline solution, Fresenius Kabi Austria Gmbh, Graz, Austria), which were subjected to three freeze and thaw cycles in liquid nitrogen and hot water respectively. They were then sonicated for 15 minutes, followed by another freeze and thaw cycle. The mixtures were then incubated overnight at 4°C, and subsequently at 37°C in a Thermoblock (Thermomixer comfort Eppendorf, Hamburg, Germany) for 30 minutes. The samples were placed into a centrifuge (Centrifuge 5424, Eppendorf, Hamburg, Germany) and rotated at 10000 rpm for 10 minutes. Finally, the supernatant was collected, and this was used with the various RDTs and ELISA.

According to the manufacturers information, for 5 microliter (µl) of blood sample QDx Malaria Pv/Pf has a sensitivity of 98.7% for P. falciparum and 99.2% for P. vivax, with a specificity of 99.6%; QDx Malaria Pan/Pf has a sensitivity of 96% for PAN malaria and 99.6% for P. falciparum, with a specificity of 99.6%. Whereas, BinaxNow® possesses, for a blood sample of 15 µl, a sensitivity of 99.7% for P. falciparum and 93.5% for P. vivax, with a specificity of 94.2% and 99.8%, respectively. Therefore, the greater sensitivity of the ODx Malaria cards, requiring less sample, perhaps make it possible for the test to identify smaller fragments of the remaining antigen, however, this could also lead to a higher false positive rate. Whereas, the greater specificity of the BinaxNow® and ELISA, perhaps mean that these tests cannot recognize any damaged antigen present in these samples. We conclude that the only way to be certain is through proteomics, and cooperation with the commercial test providers to allow us to know for certain which antigens are binding to the antibodies on the card tests. There are a number of other issues with these tests and their use on archaeological material which has yet to be addressed in pervious publications, making them, as yet, unsubstantiated indicators of malaria in the past.
