# Supplementary material for: The elusive parasite: comparing macroscopic, immunological, and genomic approaches to identifying malaria in human skeletal remains from Sayala, Egypt (third to sixth centuries AD)
Source: Archaeol Anthropol Sci. 2021 Jun 14;13(7):115. doi: 10.1007/s12520-021-01350-z (PMC8202054; doi:10.1007/s12520-021-01350-z)
Supplement: Supplementary file 2 — (DOCX 17 kb) [file 12520_2021_1350_MOESM2_ESM.docx]

Figure and Table Captions for the Supplementary Data

**Figure SA** – *Cribra orbitalia* in Cemetery CIII, Grave 8, Individual 2 – Grade 3

**Figure SB** – *Cribra orbitalia* in Cemetery CIII, Grave 59 – Grade 2

**Figure SC** – *Cribra orbitalia* in Cemetery CIII, Grave 29 – Grade 4

**Figure SD** – *Cribra orbitalia* in Cemetery CIII, Grave 20 – Grade 1

**Figure SE** – *Cribra orbitalia* in Cemetery CIII, Grave 60 – Grade 2

**Figure SF** – *Cribra orbitalia* in Cemetery CII, Grave 139 – Grade 2

**Figure SG** – *Cribra orbitalia* in Cemetery CI, Grave 47, Individual 1 – Grade 1

**Figure SH** – *Cribra orbitalia* in Cemetery CI, Grave 54, Individual 1 – Grade 2

**Figure SI** – *Cribra orbitalia* in Cemetery CI, Grave 20 – Grade 4

**Figure SJ** – *Cribra orbitalia* in Cemetery N, Grave 12 – Grade 3-4

**Figure SV: An example of the QDx Malaria card test DiaSys Pv/Pf results.** The results of the analysis of bone samples – CI/47/1 EURAC 2242, CI/54/1 EURAC 2244, both of which are positive (note the faint lines next to the Pv and Pf on the test); alongside a negative control sample from Aspern Seestadt on the left.

**Figure SW: Taxonomic overview of the sequence reads in the merged shotgun and captured datasets.** The metagenomic reads were taxonomically assigned using the Diamond tool (Buchfink, Xie, and Huson 2015) against the NCBI non-redundant protein database. The DIAMOND tables were converted to rma6 (blast2rma tool) format (--minPercentIdentity 97 –minSupport 5), imported into MEGAN6 software (Huson et al. 2016) **(A) Taxonomic Overview of the shotgun dataset of sample 2229.** The visualization has been done using the Krona tool (Ondov, Bergman, and Phillippy 2011). **(B) General distribution of the taxonomically assigned reads of the merged shotgun and captured datasets of all samples to the three main kingdoms and to the genus *Plasmodium*.** The number of reads assigned to the genus *Plasmodium* is provided below the bar charts. For details to the sample IDs displayed in the legend please refer to the Supplementary Table S1.

**Figure SX: Analysis of the human mitochondrial DNA of Individual 139 (EURAC ID 2240). (A) Ancient DNA damage profiling using mapDamage2.** The human mitochondrial reads display in increased C to T substitution frequency at the 5´read end indicative for ancient DNA. **(B) Mitochondrial haplogroup assignment using HaplGrep2.** Tree including all related polymorphisms relative to the rCRS.

**Figure SY: BLAST results for the reads identified as *Plasmodium***. All reads that initially mapped against the full *Plasmodium* assemblies and that were taxonomically assigned to the genus *Plasmodium* by the blastN and MEGAN6 analysis were once more subjected to a manual blastN analysis against the complete NCBI-nt database. Displayed in black are always the first eight hits of eight reads that have as top hit a *Plasmodium*-like sequence. In red we show in addition a blast a hit, that displays like other reads as a first hit a taxon different to *Plasmodium*. This hit is followed by subsequent hits of *Plasmodium*- like sequences that have a lower E-value.

**Figure SZ: Gene coverage and distribution of the enriched sequence reads mapped onto the 5,967 bp large reference mitochondrial genome of *Plasmodium falciparum* 3D7 (LR605957.1).** The coverage plots of the studied samples with captured reads (2229C, 2239C, 2240C, 2247C, 2248C) and previously published control samples (SRR4425648, SRR4425649) are displayed in different colors superimposed onto the genomic sequence. Below the coverage plots is the GC content and the gene annotation of the modern reference provided. Genes highlighted in red contain regions where read stacking occurs.

**Table S1:** Shotgun and capture (in red) sequencing output and mapping data against human and Plasmodia reference genomes.
