# Supplementary figures and images for: The elusive parasite: comparing macroscopic, immunological, and genomic approaches to identifying malaria in human skeletal remains from Sayala, Egypt (third to sixth centuries AD)
Source: Archaeol Anthropol Sci. 2021 Jun 14;13(7):115. doi: 10.1007/s12520-021-01350-z (PMC8202054; doi:10.1007/s12520-021-01350-z)

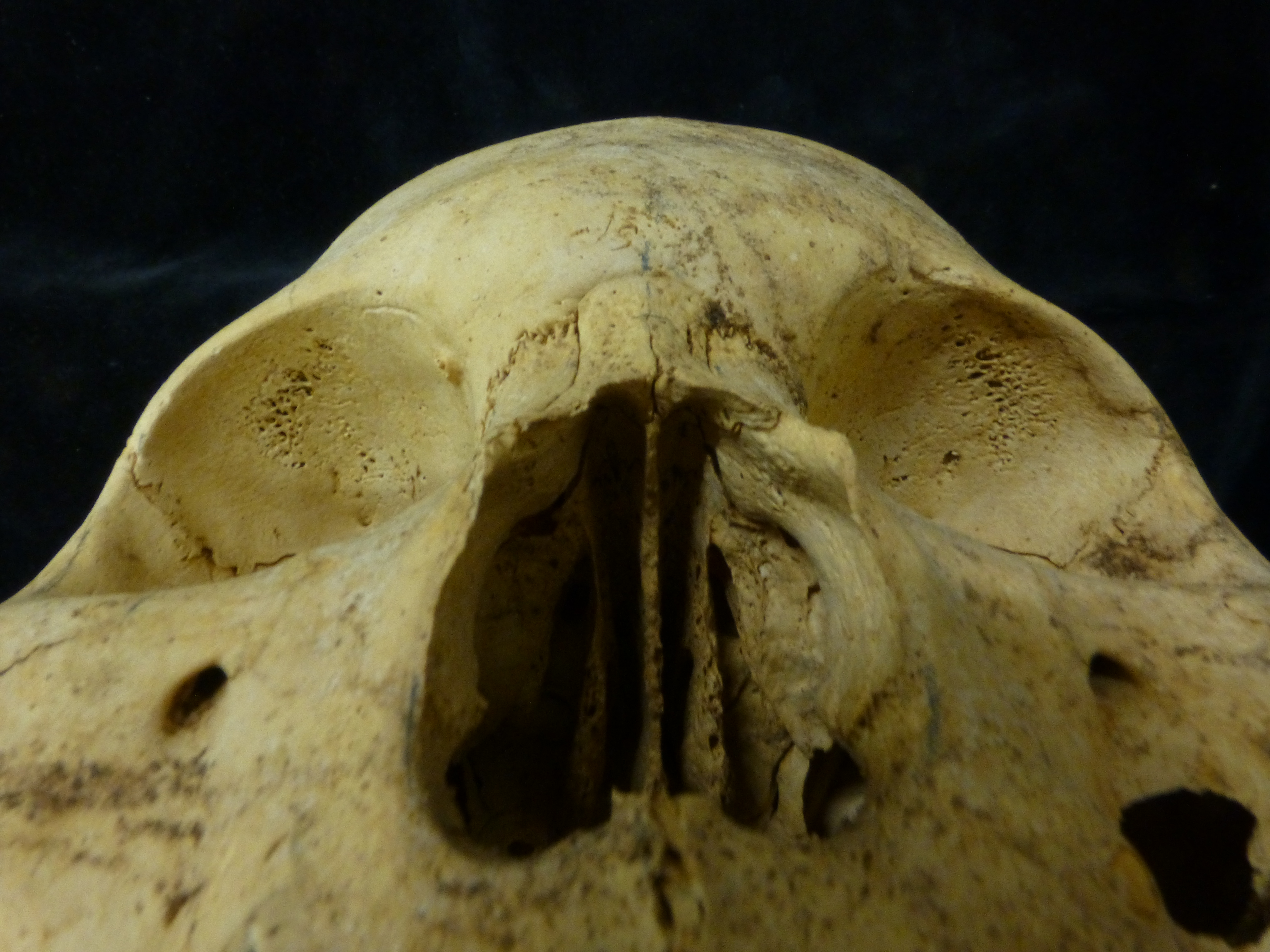

Supplement: Supplementary file 4 — (JPG 5321 kb) [file 12520_2021_1350_MOESM4_ESM.jpg]

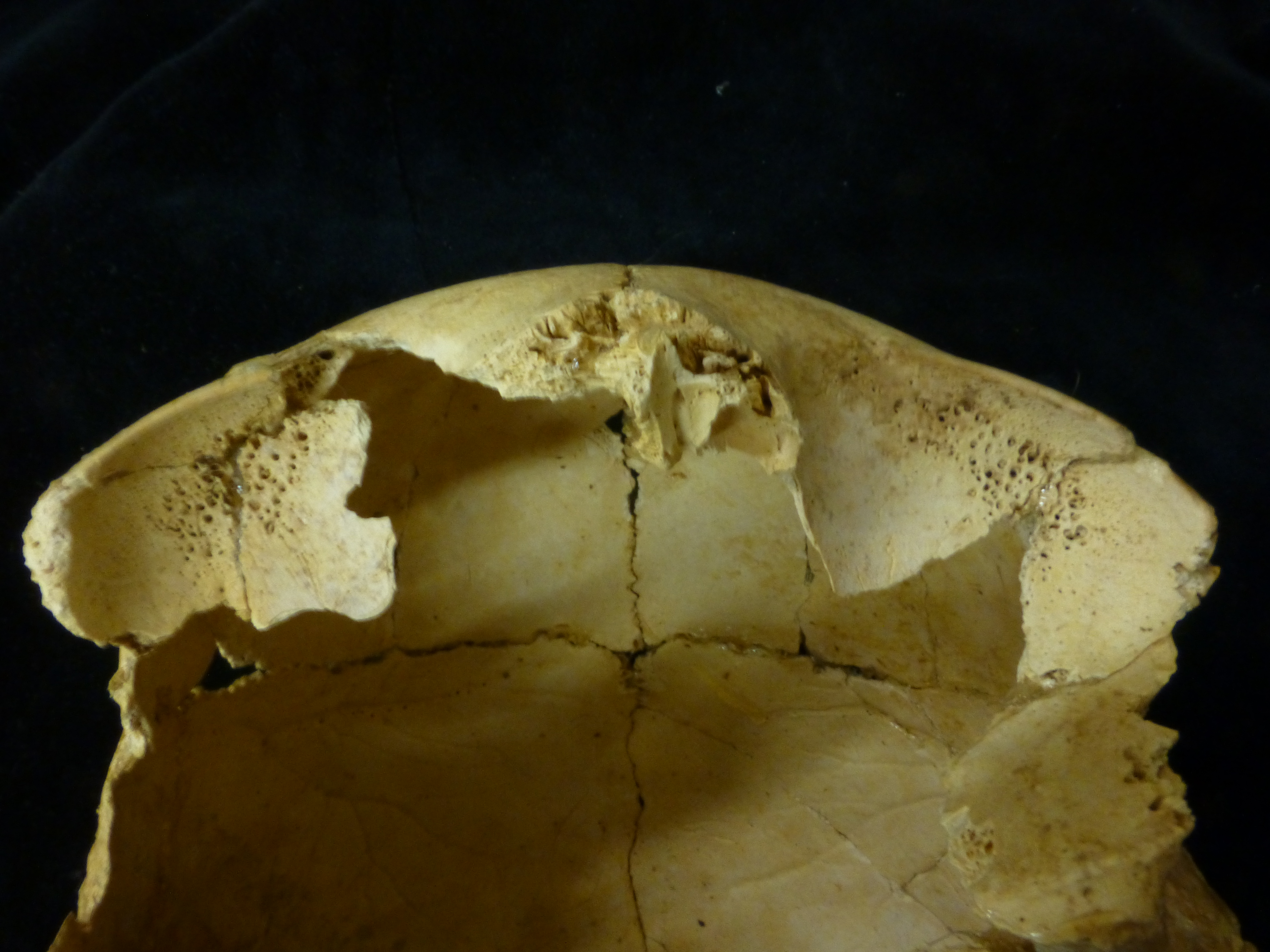

Supplement: Supplementary file 5 — (JPG 4632 kb) [file 12520_2021_1350_MOESM5_ESM.jpg]

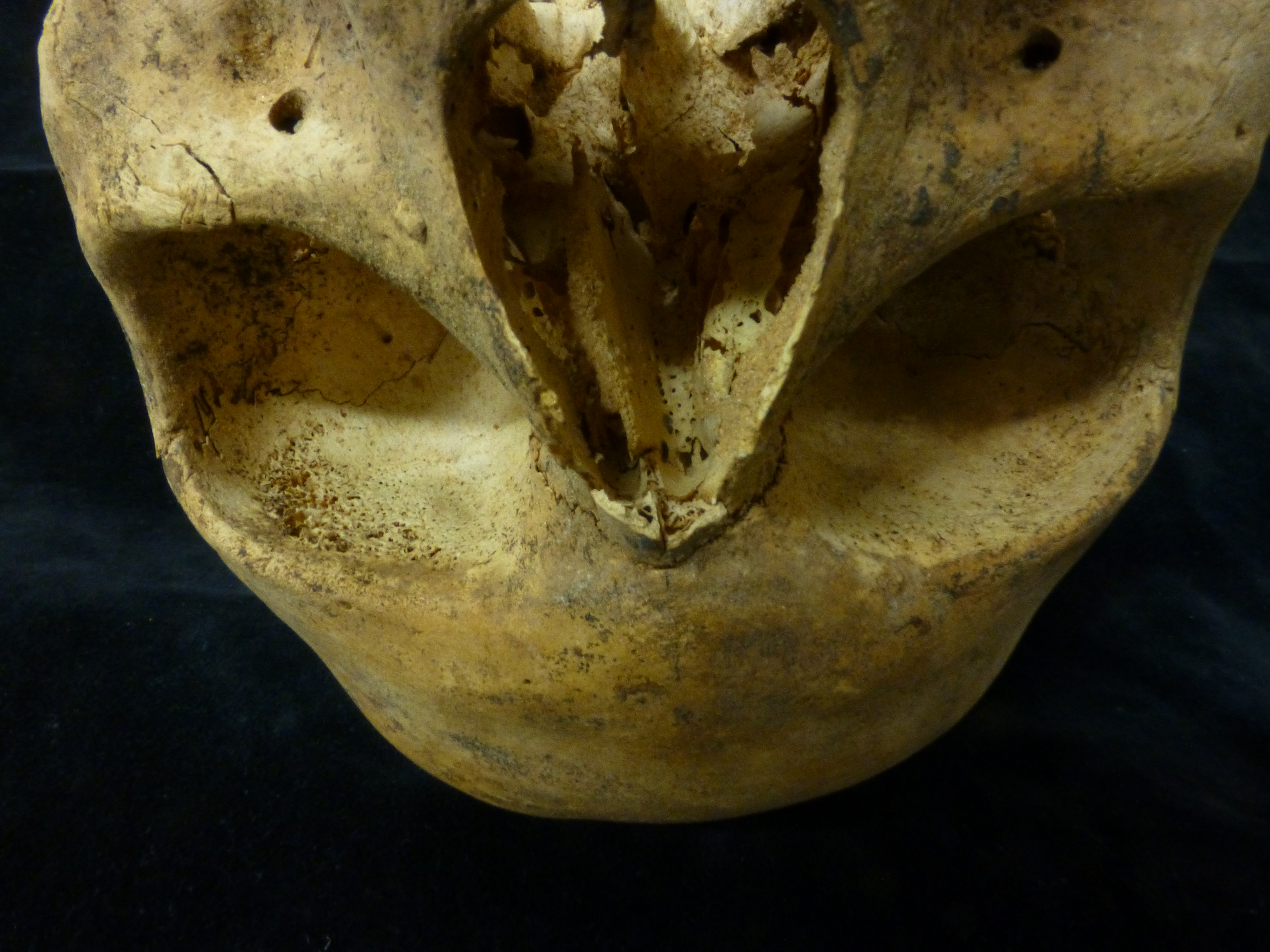

Supplement: Supplementary file 6 — (JPG 5698 kb) [file 12520_2021_1350_MOESM6_ESM.jpg]

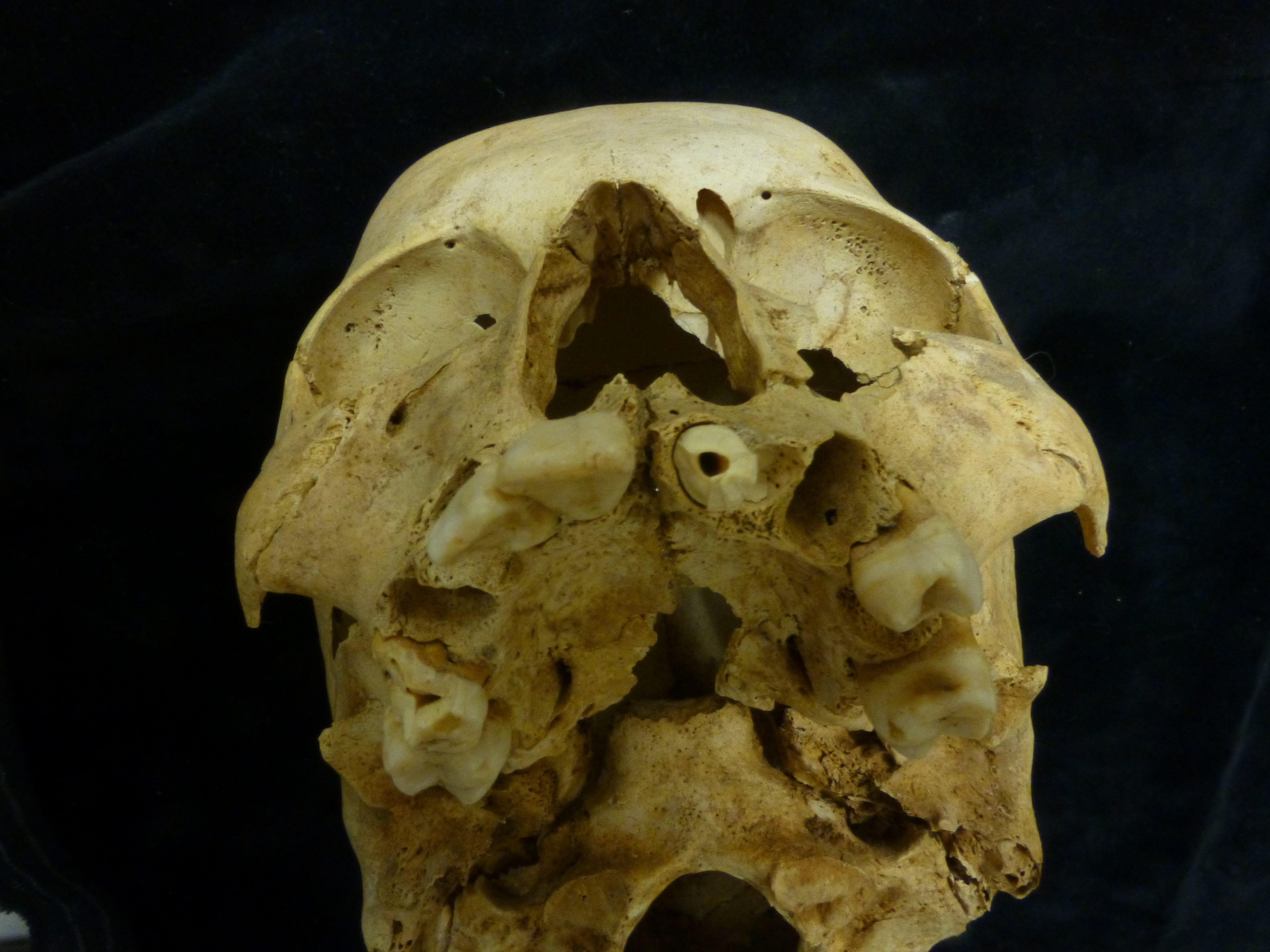

Supplement: Supplementary file 7 — (JPG 5139 kb) [file 12520_2021_1350_MOESM7_ESM.jpg]

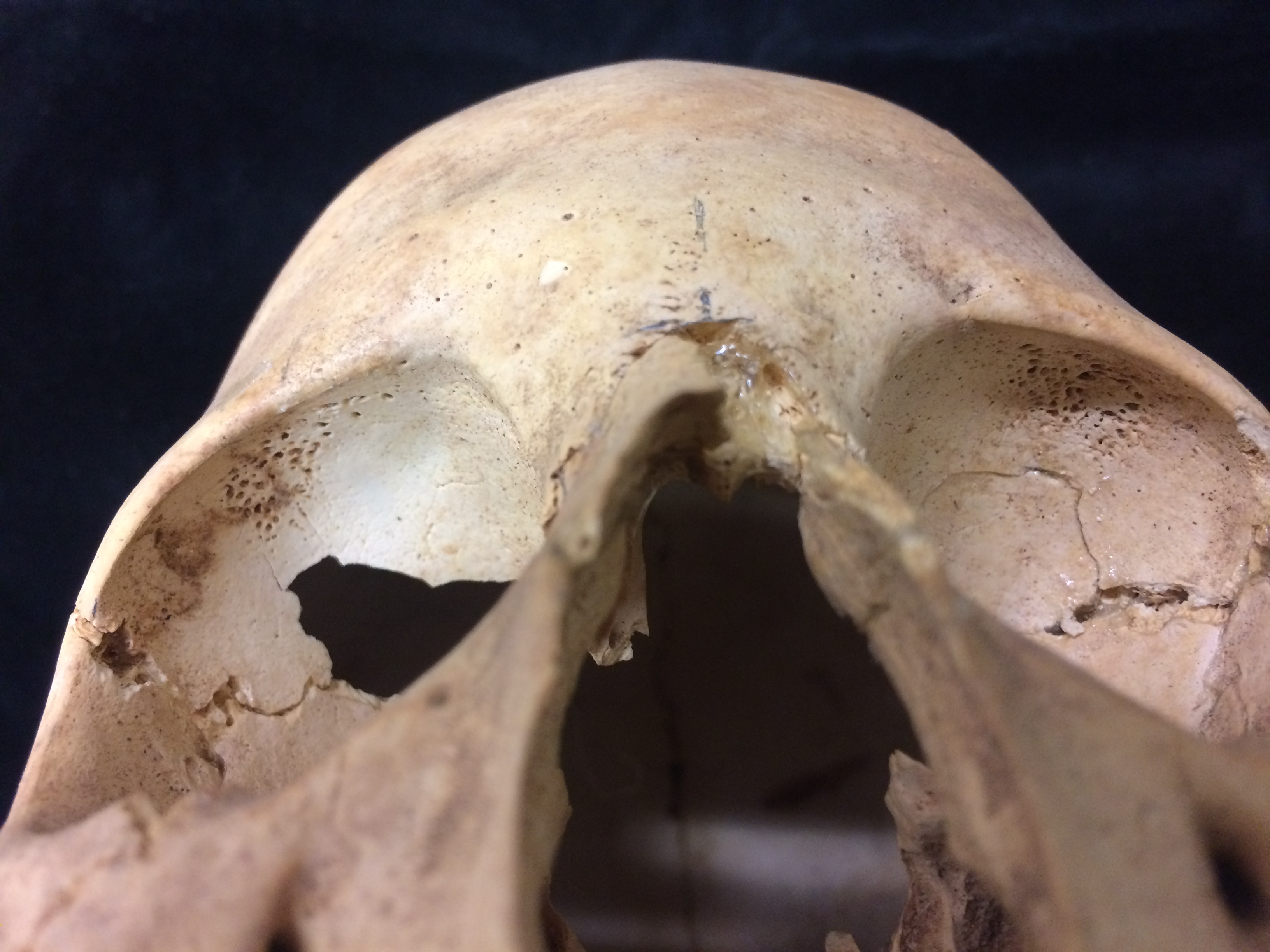

Supplement: Supplementary file 8 — (JPG 1334 kb) [file 12520_2021_1350_MOESM8_ESM.jpg]

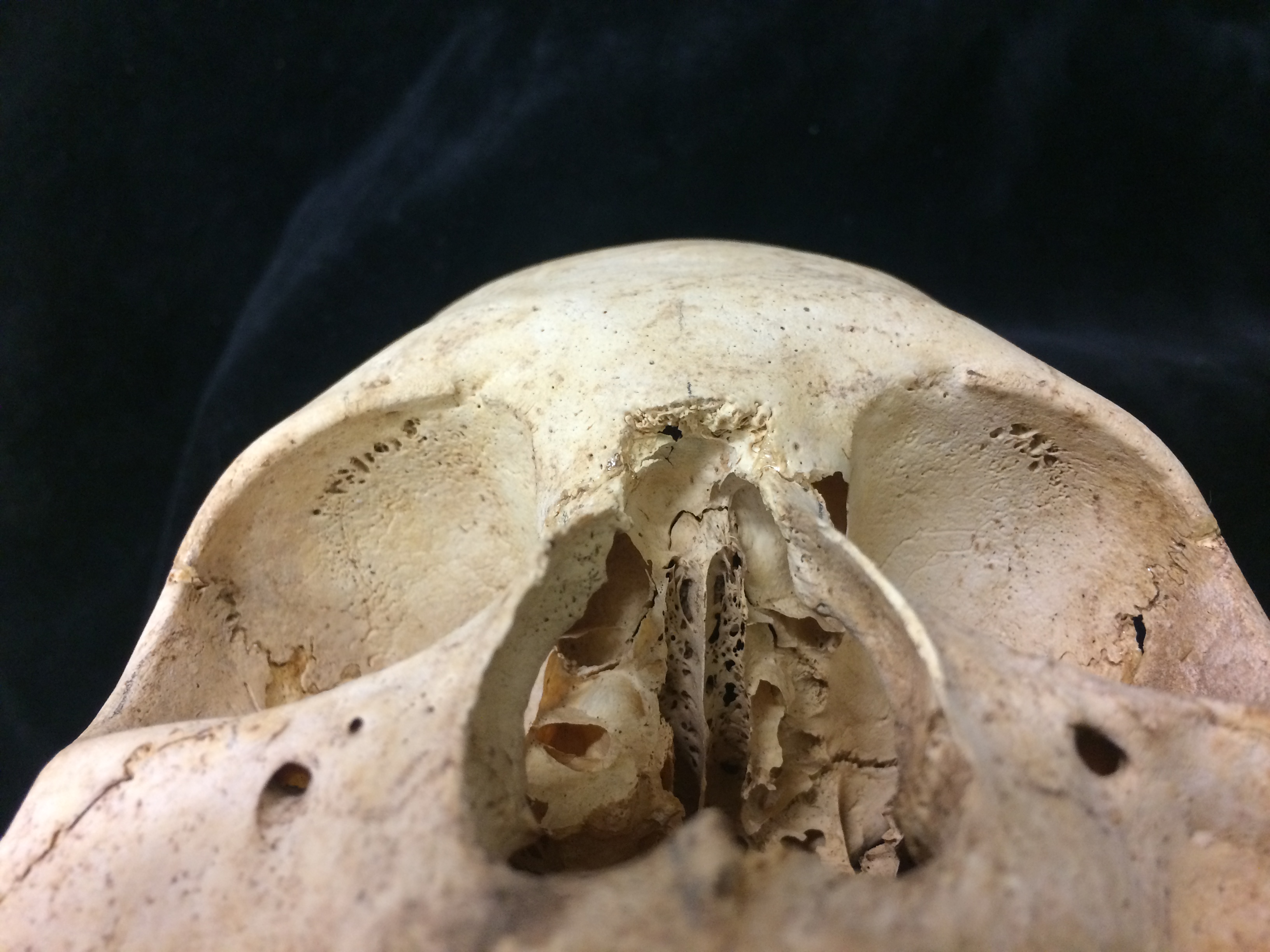

Supplement: Supplementary file 9 — (JPG 1363 kb) [file 12520_2021_1350_MOESM9_ESM.jpg]

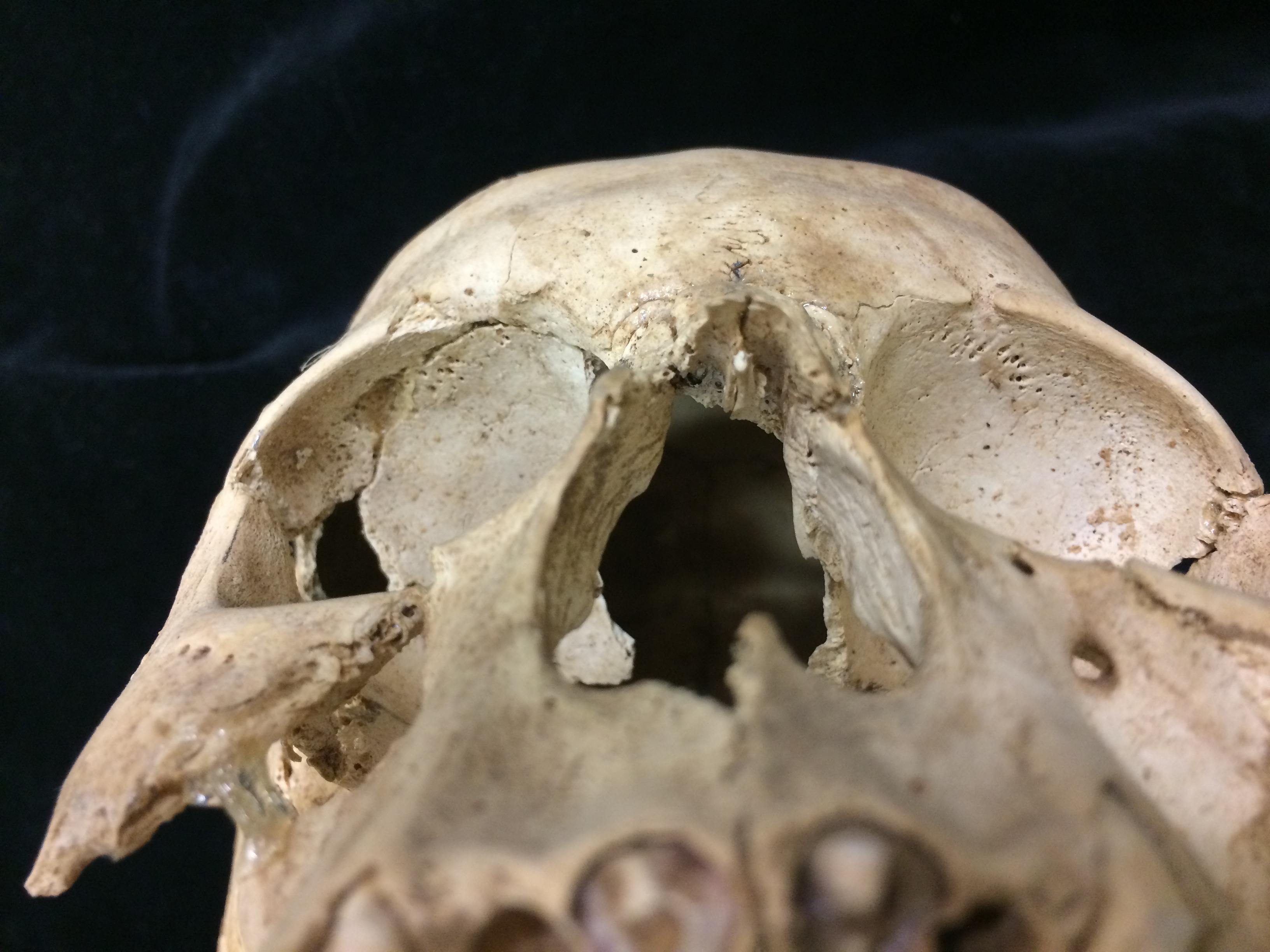

Supplement: Supplementary file 10 — (JPG 1349 kb) [file 12520_2021_1350_MOESM10_ESM.jpg]

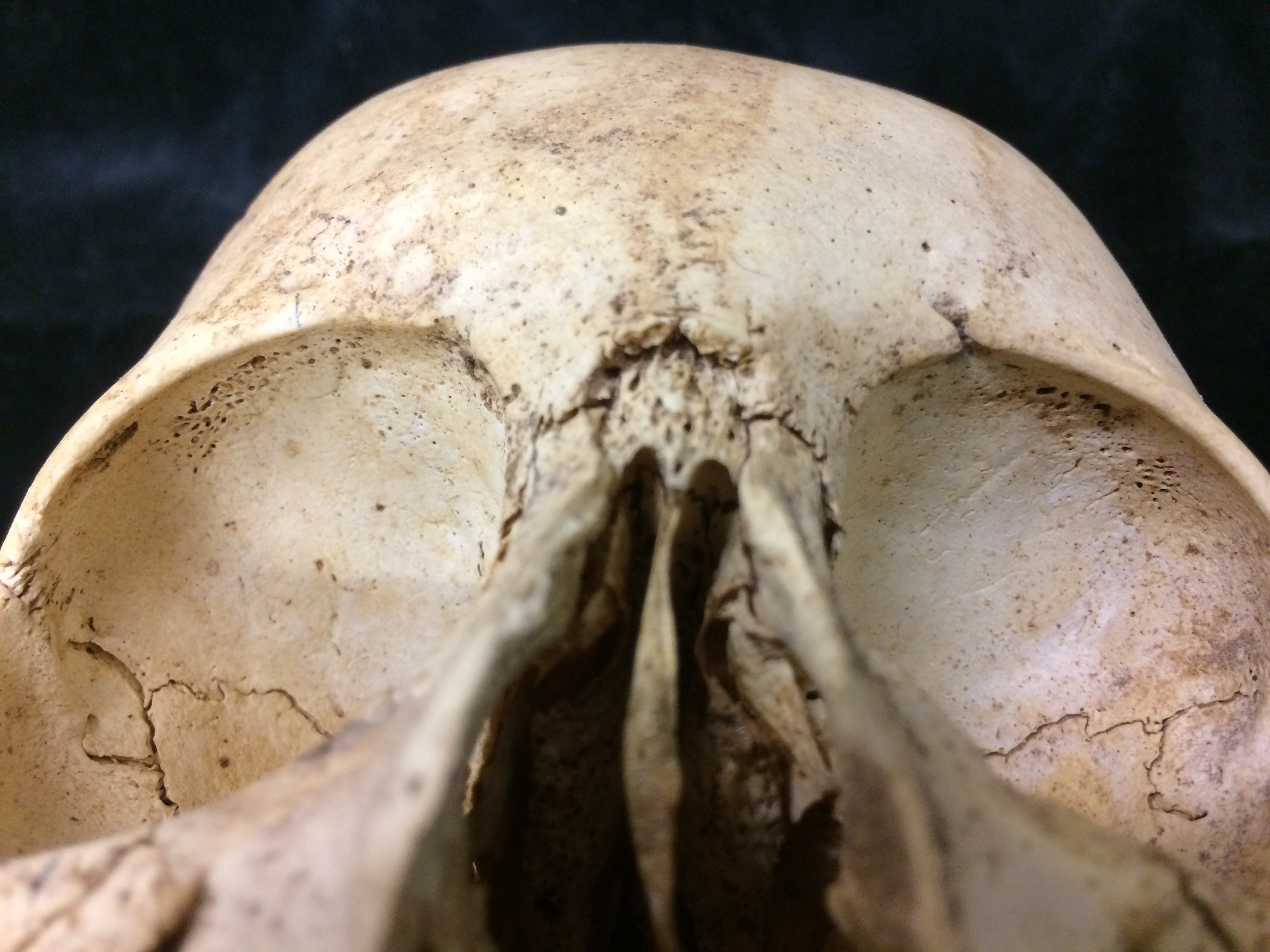

Supplement: Supplementary file 11 — (JPG 1028 kb) [file 12520_2021_1350_MOESM11_ESM.jpg]

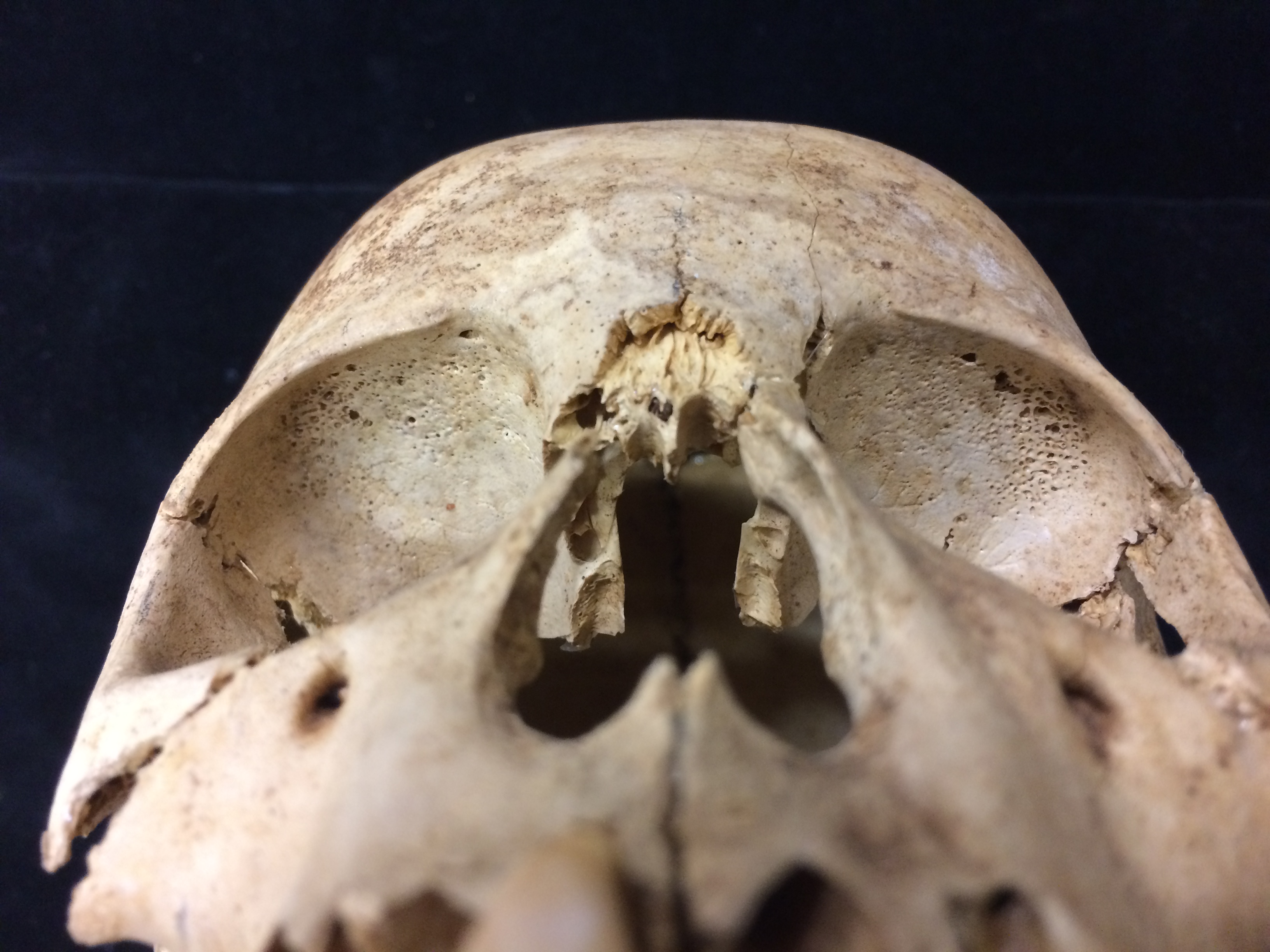

Supplement: Supplementary file 12 — (JPG 1341 kb) [file 12520_2021_1350_MOESM12_ESM.jpg]

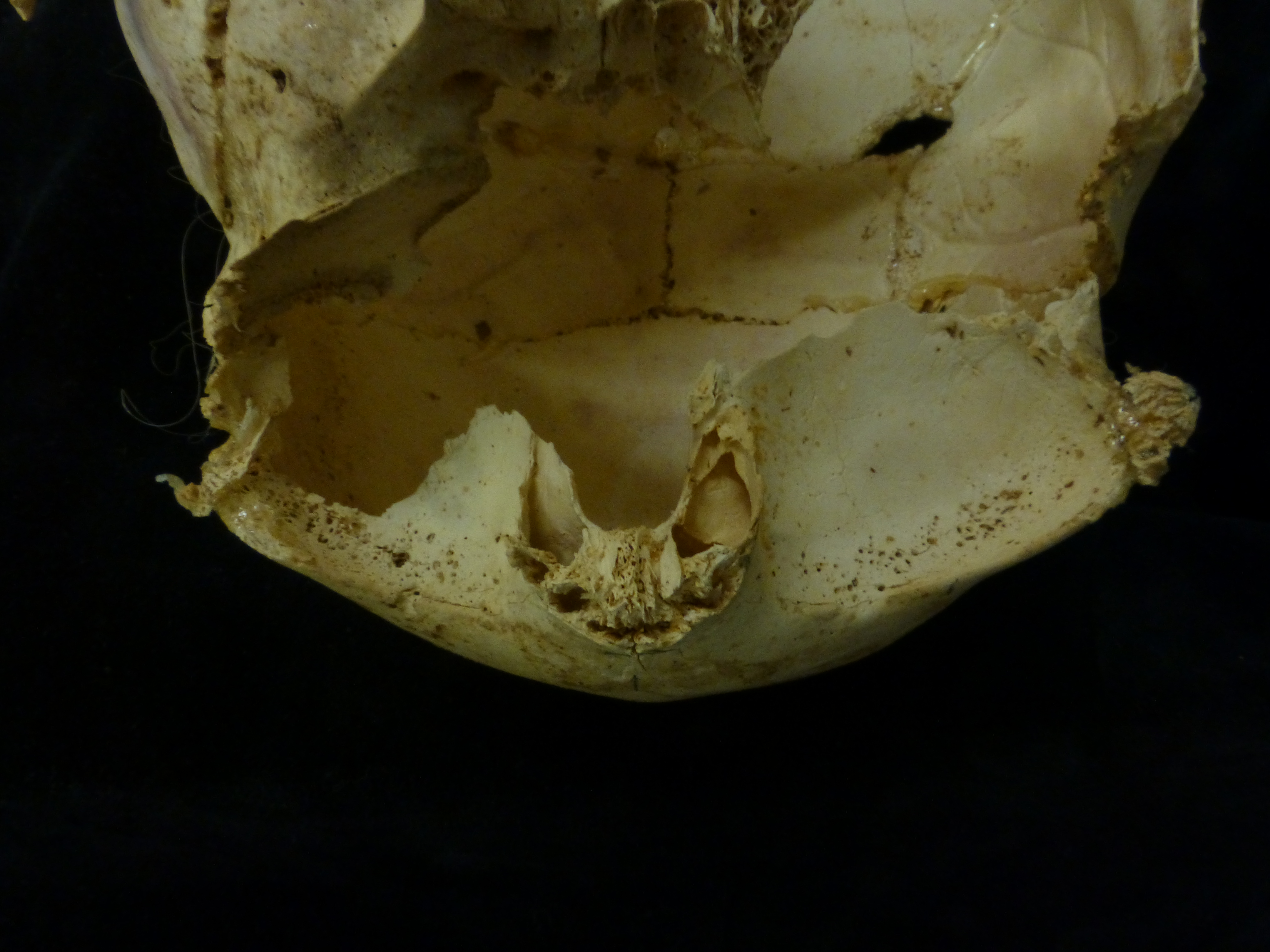

Supplement: Supplementary file 13 — (JPG 4192 kb) [file 12520_2021_1350_MOESM13_ESM.jpg]

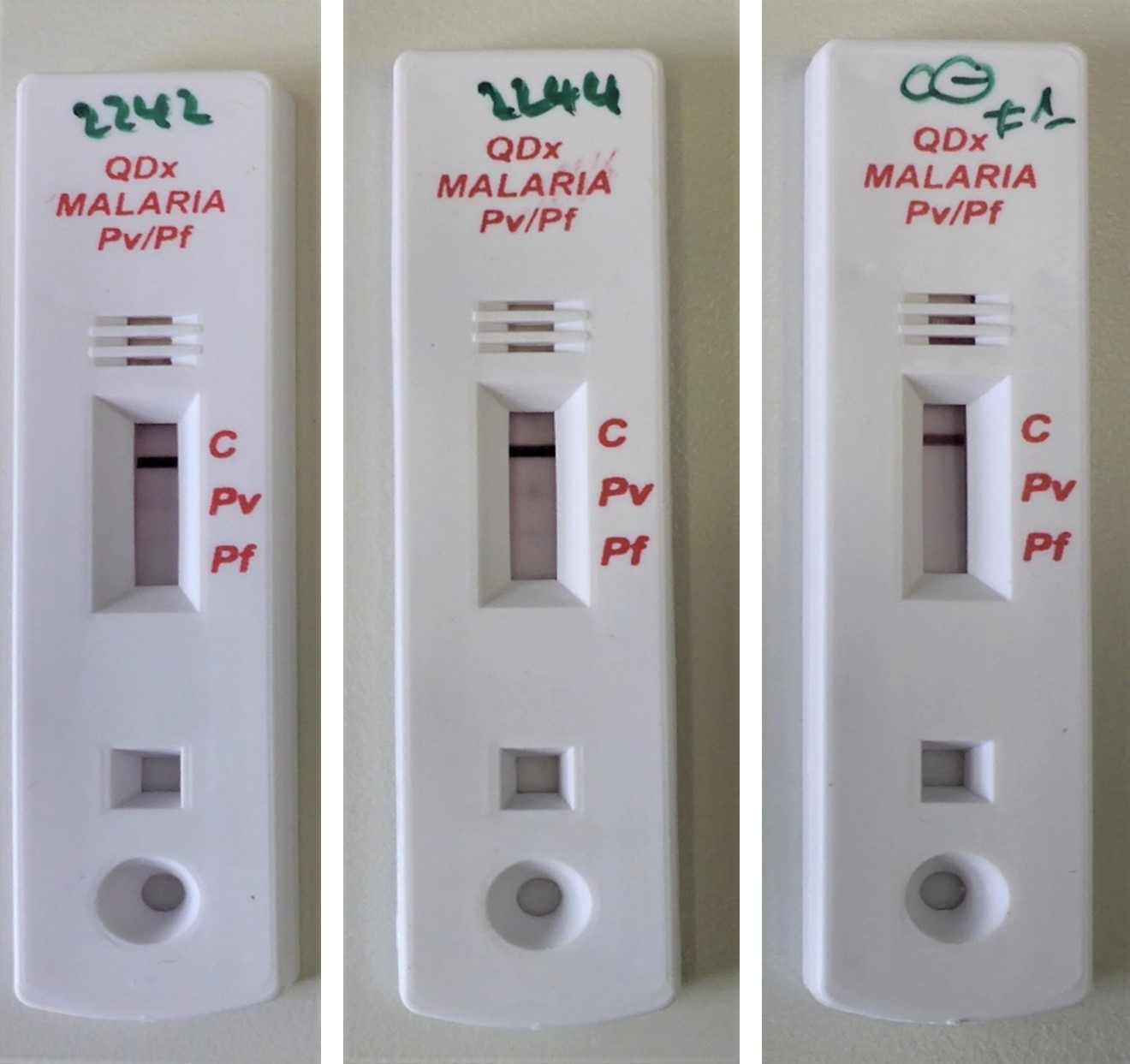

Supplement: Supplementary file 14 — (JPG 174 kb) [file 12520_2021_1350_MOESM14_ESM.jpg]

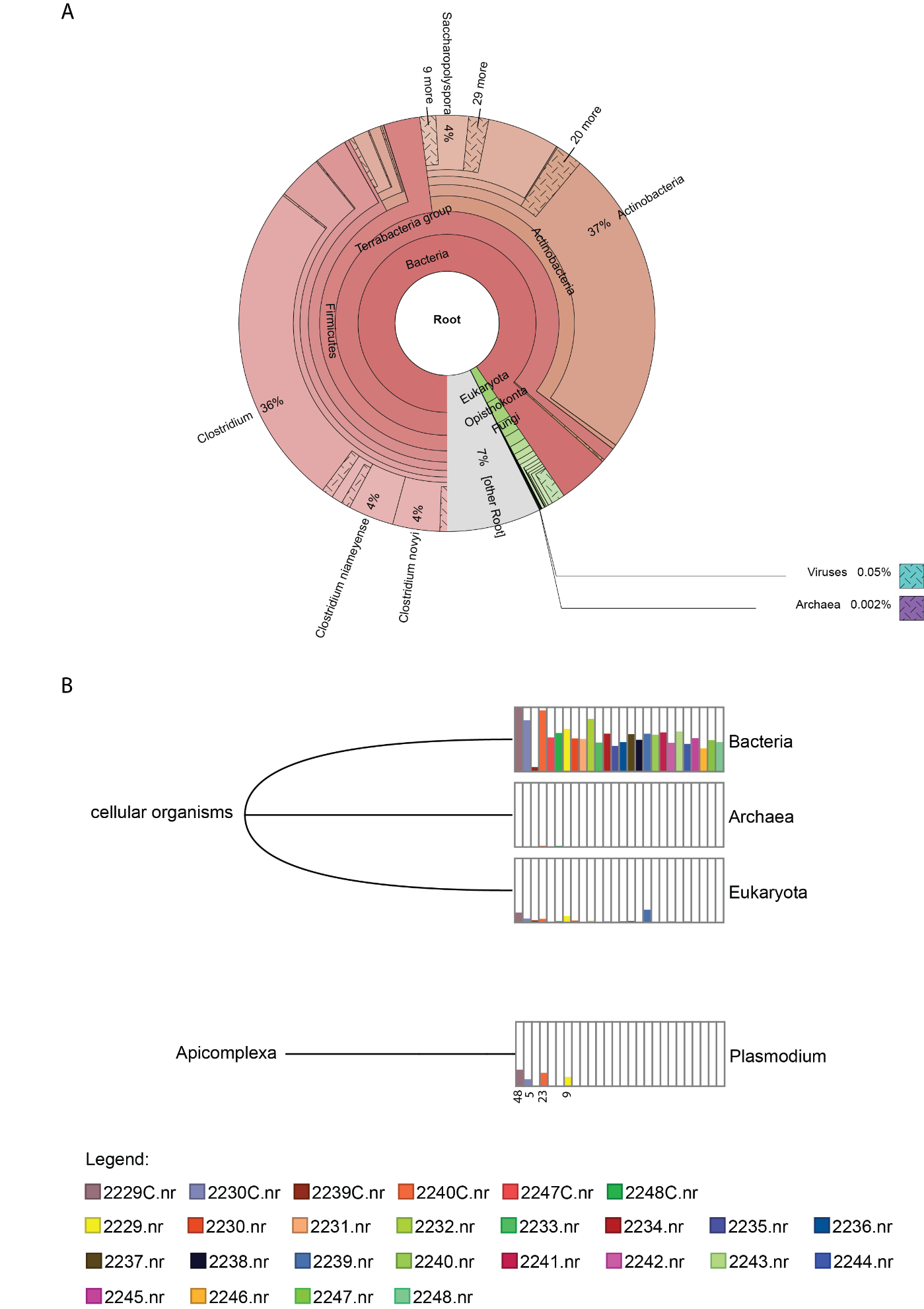

Supplement: Supplementary file 15 — (DOCX 270 kb) [file 12520_2021_1350_MOESM15_ESM.docx]

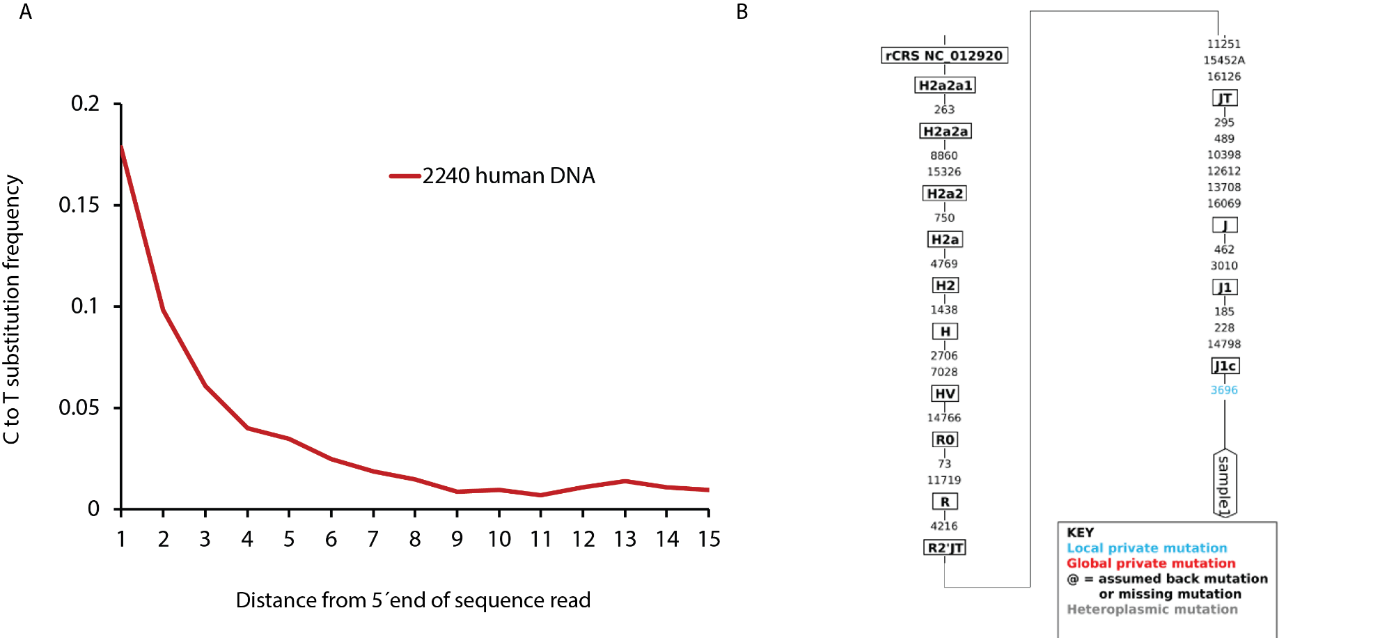

Supplement: Supplementary file 16 — (DOCX 120 kb) [file 12520_2021_1350_MOESM16_ESM.docx]

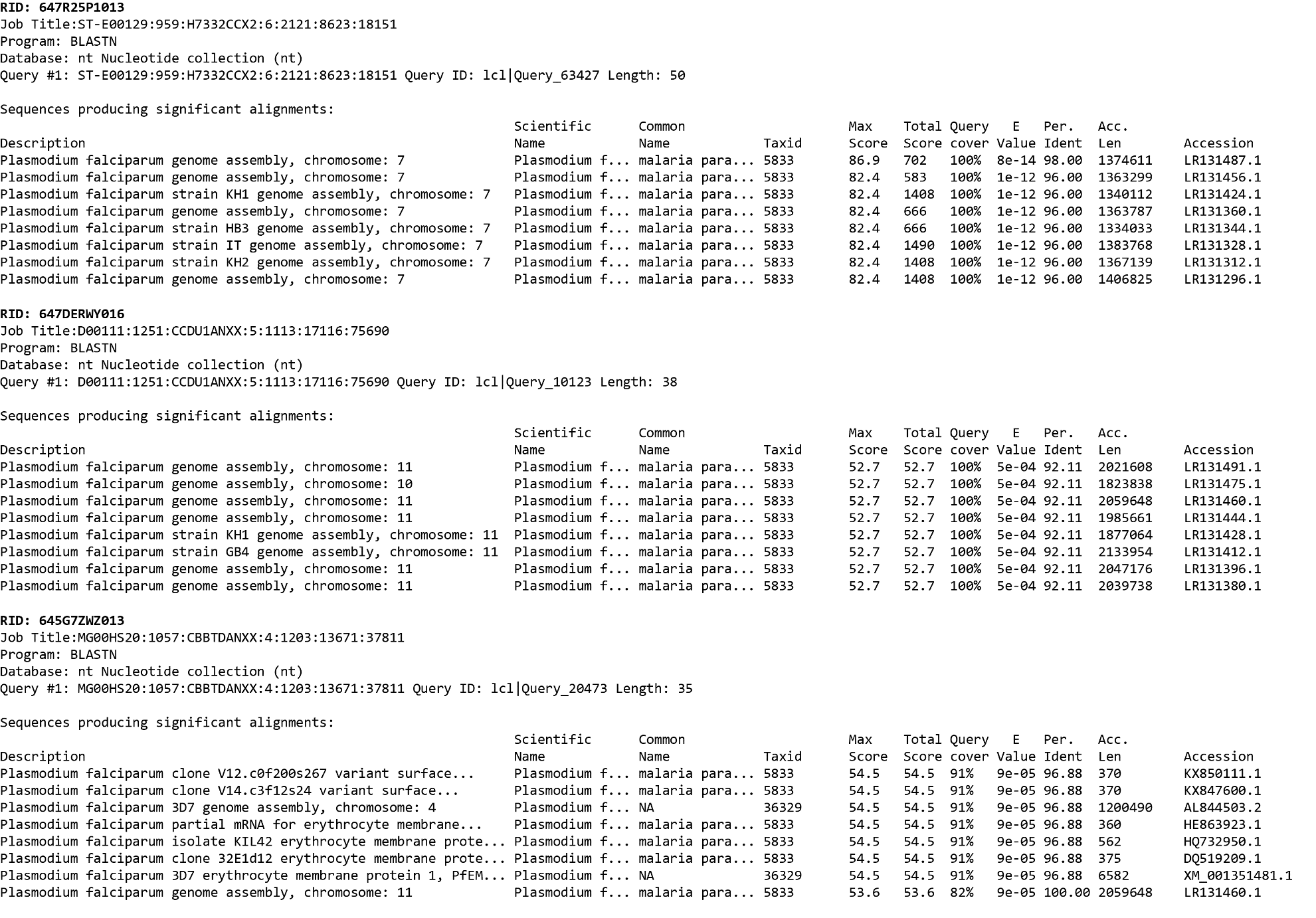


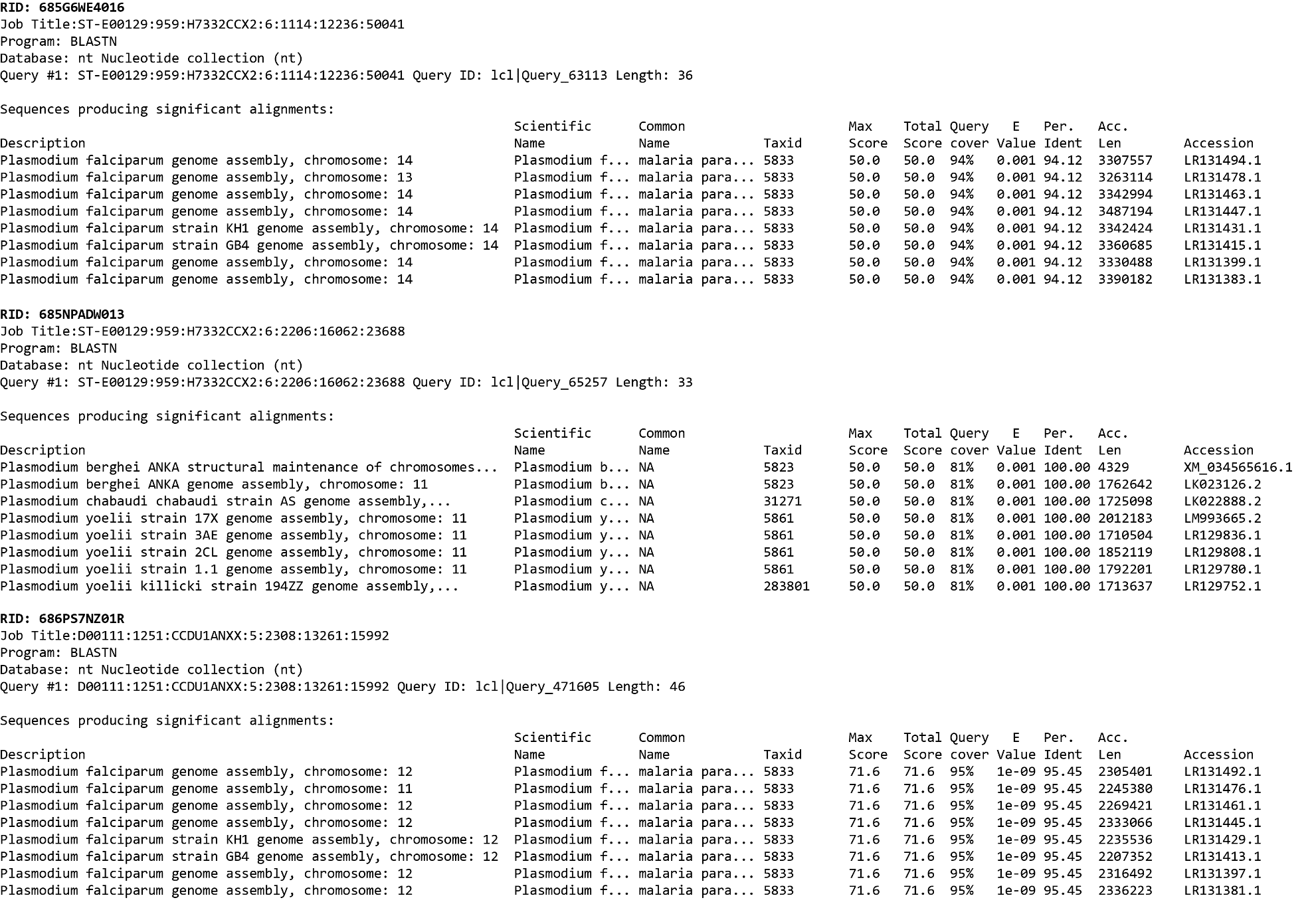


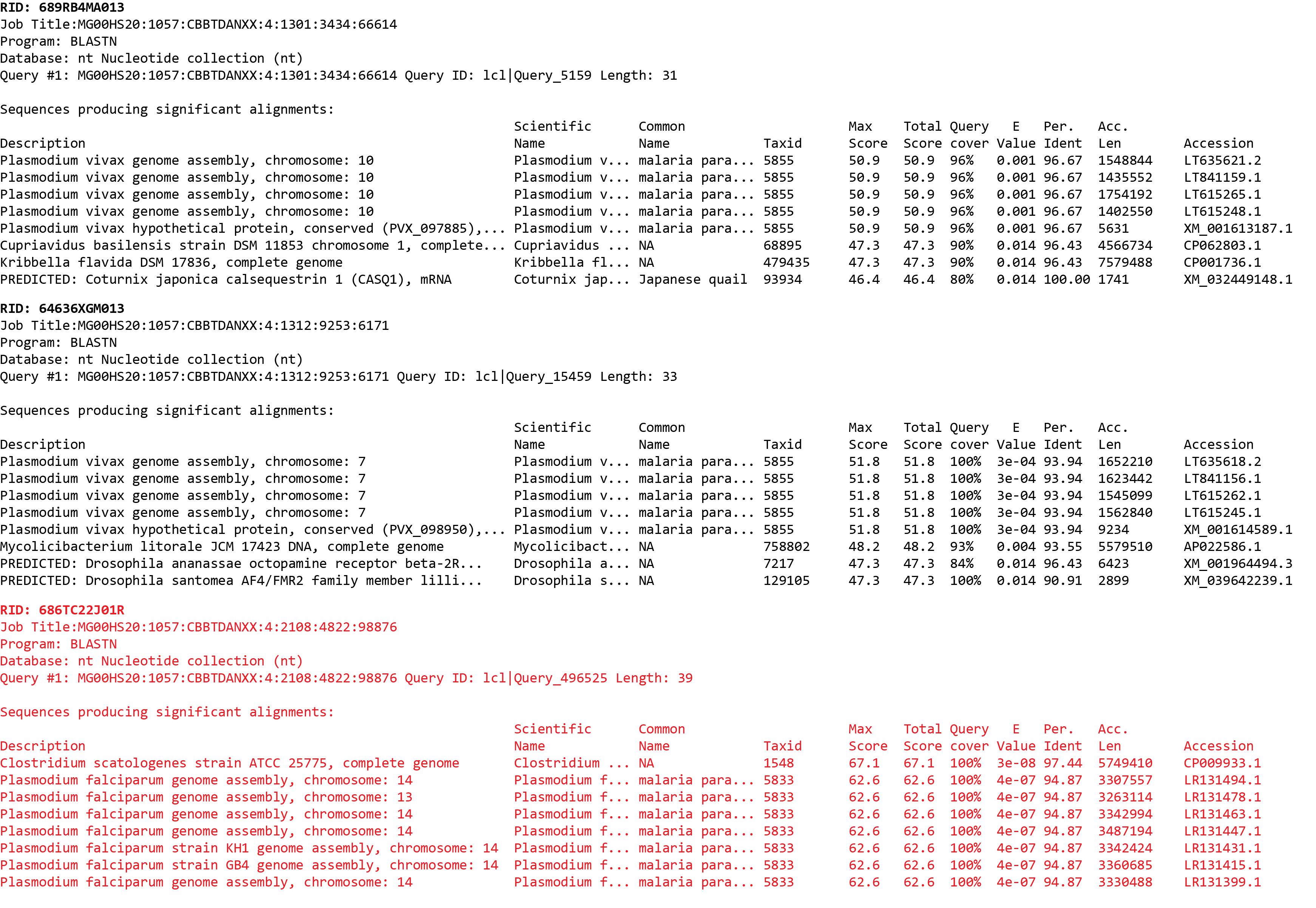

Supplement: Supplementary file 17 — (DOCX 823 kb) [file 12520_2021_1350_MOESM17_ESM.docx]

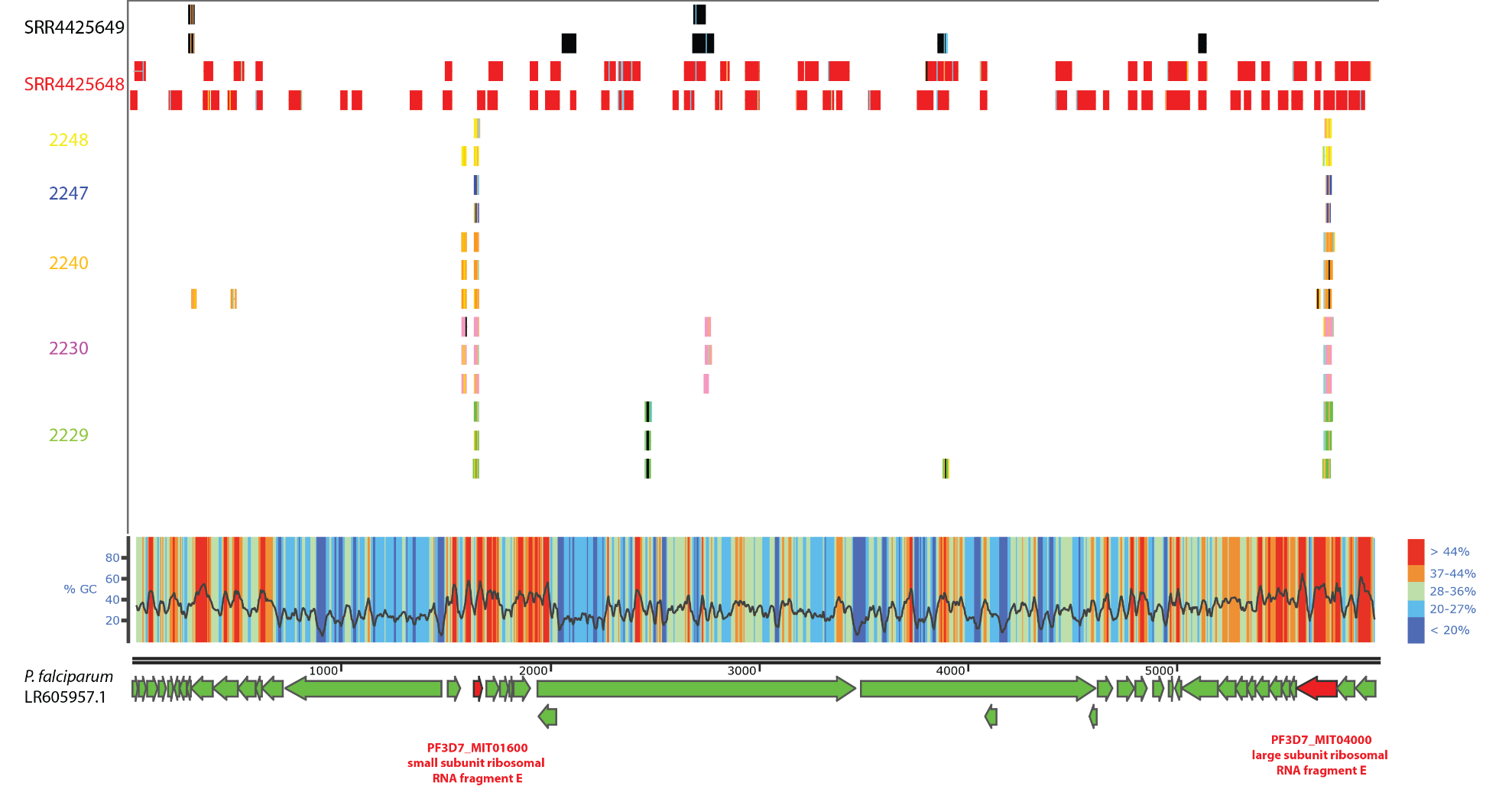

Supplement: Supplementary file 18 — (DOCX 166 kb) [file 12520_2021_1350_MOESM18_ESM.docx]
